# Artificial-intelligence-driven discovery of catalyst *genes* with application to CO_2_ activation on semiconductor oxides

**DOI:** 10.1038/s41467-022-28042-z

**Published:** 2022-01-20

**Authors:** Aliaksei Mazheika, Yang-Gang Wang, Rosendo Valero, Francesc Viñes, Francesc Illas, Luca M. Ghiringhelli, Sergey V. Levchenko, Matthias Scheffler

**Affiliations:** 1 The NOMAD Laboratory at the Fritz-Haber-Institut der Max-Planck-Gesellschaft, 14195 Berlin-Dahlem, Germany; 2 Department of Chemistry and Guangdong Provincial Key Laboratory of Catalysis, Southern University of Science and Technology, 518055 Shenzhen, Guangdong China; 3 Departament de Ciència de Materials i Química Física and Institut de Química Teòrica i Computacional (IQTCUB), Universitat de Barcelona, c/ Martí i Franquès 1, Barcelona, 08028 Spain; 4 Zhejiang Huayou Cobalt Co.,Ltd., No. 18 Wuzhen East Road, Tongxiang Economic Development Zone, 314500 Jiaxing, Zhejiang China; 5 The NOMAD Laboratory at the Humboldt University of Berlin, 12489 Berlin, Germany; 6 Skolkovo Institute of Science and Technology, Skolkovo Innovation Center, Bolshoy Boulevard 30, bld. 1, 121205 Moscow, Russia

**Keywords:** Materials for energy and catalysis, Theory and computation, Atomistic models, Computational chemistry

## Abstract

Catalytic-materials design requires predictive modeling of the interaction between catalyst and reactants. This is challenging due to the complexity and diversity of structure-property relationships across the chemical space. Here, we report a strategy for a rational design of catalytic materials using the artificial intelligence approach (AI) subgroup discovery. We identify catalyst *genes* (features) that correlate with mechanisms that trigger, facilitate, or hinder the activation of carbon dioxide (CO_2_) towards a chemical conversion. The AI model is trained on first-principles data for a broad family of oxides. We demonstrate that surfaces of experimentally identified good catalysts consistently exhibit combinations of *genes* resulting in a strong elongation of a C-O bond. The same combinations of *genes* also minimize the OCO-angle, the previously proposed indicator of activation, albeit under the constraint that the Sabatier principle is satisfied. Based on these findings, we propose a set of new promising catalyst materials for CO_2_ conversion.

## Abstract (web summary)

Here the authors demonstrate an artificial-intelligence based approach to identify catalytic materials features that correlate with mechanisms that trigger, facilitate, or hinder CO2 catalytic reactions.

## Introduction

The need for converting stable molecules such as carbon dioxide (CO_2_), methane, or water into useful chemicals and fuels is growing quickly along with the depletion of fossil-fuel reserves and the pollution of the environment^1–3^. Such a conversion does not have a satisfactory solution, so far. In particular, CO_2_ conversion remains one of the most important societal and technological challenges^1,2,4–8^.

The general understanding in heterogeneous catalysis is that a stable molecule such as CO_2_ needs to be “prepared” before its catalytic conversion occurs. This leads to the notion of molecular activation^9^. However, on one hand, this notion encompasses a very wide variety of processes (adsorption, photo-excitation, application of electric field, etc.) and materials (including compositional and structural variability), and it remains unclear which properties of the catalytic material and the adsorbed molecule determine the final chemistry, what is the relationship between the two sets of properties, and how general this relationship may be. On the other hand, finding the set of descriptive parameters of a catalytic material that characterize the catalytic performance in a particular process, or even in general for a given reactant, would be very valuable, because it would allow us to quickly search for promising candidate catalysts using rational design^10–17^. We call these properties materials *genes*. The *genes* do not necessarily correlate with catalytic activity by themselves. Similar to biological genes, their role depends on the combination in which they occur, and can be either beneficial or detrimental to the catalytic activity.

Several strategies exist to find such properties for a given reaction. One way is to explore the free-energy surface for each catalyst candidate, which is a slow and resource-consuming process, and currently computationally unfeasible for many materials on a high-throughput basis. An alternative approach consists in searching for a correlation between experimentally determined material’s properties and its catalytic performance. Such a strategy requires consistent experimental measurements at well-defined conditions for a set of materials. To the best of our knowledge, such consistent data have not been reported so far for CO_2_ conversion on semiconductor oxides. Moreover, available publications usually do not report unsuccessful experimental results. These issues and a strategy to address them have been recently discussed in our publication^18^.

Yet another strategy is to find an indicator of activation, namely, a property of the system that directly indicates the certain catalytic performance of the material^10^. Indicators are distinguished from materials *genes* based on a qualitatively different level of computational complexity. The indicator can still be unfeasible or hard for a high-throughput study of hundreds of thousands or millions of materials. However, when it can be calculated for a few tens or hundreds of materials in a reasonable time, these data can then be used to find materials *genes* that control the value of the indicator. Since a direct search for a relationship between the indicator and catalytic performance of material would also require a consistent set of data of turnover frequency (TOF), selectivity, and yield values, one could instead consider several most promising indicators, find out which materials are good catalysts, and then check which indicators correlate with this observation. This approach also addresses the problem of defining activation in terms of the adsorbed-molecule properties as potential indicators of catalytic activity.

Catalytic conversion of CO_2_ requires activation of other reactants as well, e.g., molecular hydrogen, water, or methane. In particular, hydrogen can serve as an environmentally friendly reagent that can be produced by water electrolysis or photo-splitting avoiding extra CO_2_ emissions^19–21^. Also, oxygen vacancies have been proposed as active sites for CO_2_ conversion on some materials^22^. Therefore, predictions of catalytic activity of materials for CO_2_ conversion can be refined based on analysis of activation of other reactants and defects. An additional challenge is to ensure that the useful products, as well as the surface catalytic activity, are preserved under the conditions of activation and subsequent conversion. While the strong C–O double bonds in CO_2_ can be weakened or even broken by adsorption at a solid surface at an elevated temperature, this may also lead to too strong adsorption or further dissociation of the molecule, so that the catalytic surface is poisoned by carbonate or carbon deposits. Weak adsorption, on the other hand, means no activation.

In this work, we combine first-principles calculations with an artificial-intelligence (AI) method, subgroup discovery (SGD), to identify pristine materials properties that optimize indicators of catalytic CO_2_ activation. Moreover, SGD allows identifying one or more distinct combinations of materials features (*genes*) that promote activation. We focus on oxide materials as candidate catalysts. Oxides are structurally and compositionally stable under realistic temperatures and can be less expensive than the traditional precious metal-containing catalysts^23–25^. Activation of other reactants and defects are not considered. As shown below, meaningful predictions can be made based solely on the analysis of the adsorption properties of CO_2_ on pristine surfaces. This confirms that these properties are good indicators of activation with a viable optimization pathway at least for the chosen class of materials. The Sabatier principle is taken into account by ensuring that the adsorption energy is not too large or too small. In order to ensure reproducibility of our AI data analysis, we provide all necessary metadata (input parameters) and workflow in the easily accessible form of a Jupyter notebook^26^. We argue that, with the ever-growing importance and complexity of AI, such detailed and tutorial documentation is a necessity of good scientific practice. Our approach is applicable to a wider class of materials and molecules, not limited to oxides or CO_2_. Our study by no means encompasses all possible mechanisms of CO_2_ conversion on oxide surfaces, but it offers a clear design path among many possible ones.

## Results

### CO_2_ activation

We find that on semiconductor oxide surfaces CO_2_ is chemisorbed exclusively when the carbon atom binds to surface O-atoms. All other minima of the potential-energy surface are found to be either metastable or correspond to physisorption. Therefore, there are as many different potential chemisorption sites as there are unique O-atoms at the surface. The dataset includes all non-equivalent surface O-atoms on the 141 considered surfaces of 71 materials, which sum up to 255 unique adsorption sites. Among these sites on about 4% (10 out of 255) CO_2_ prefers to physisorb, i.e., any chemisorbed state is metastable with respect to the physisorbed one. The physisorption can be easily identified by an almost linear geometry of the adsorbed molecule, and a C–O bond distance very close to the C–O bond length in a gas-phase CO_2_ molecule, 1.17 Å.

**Fig. 1:**
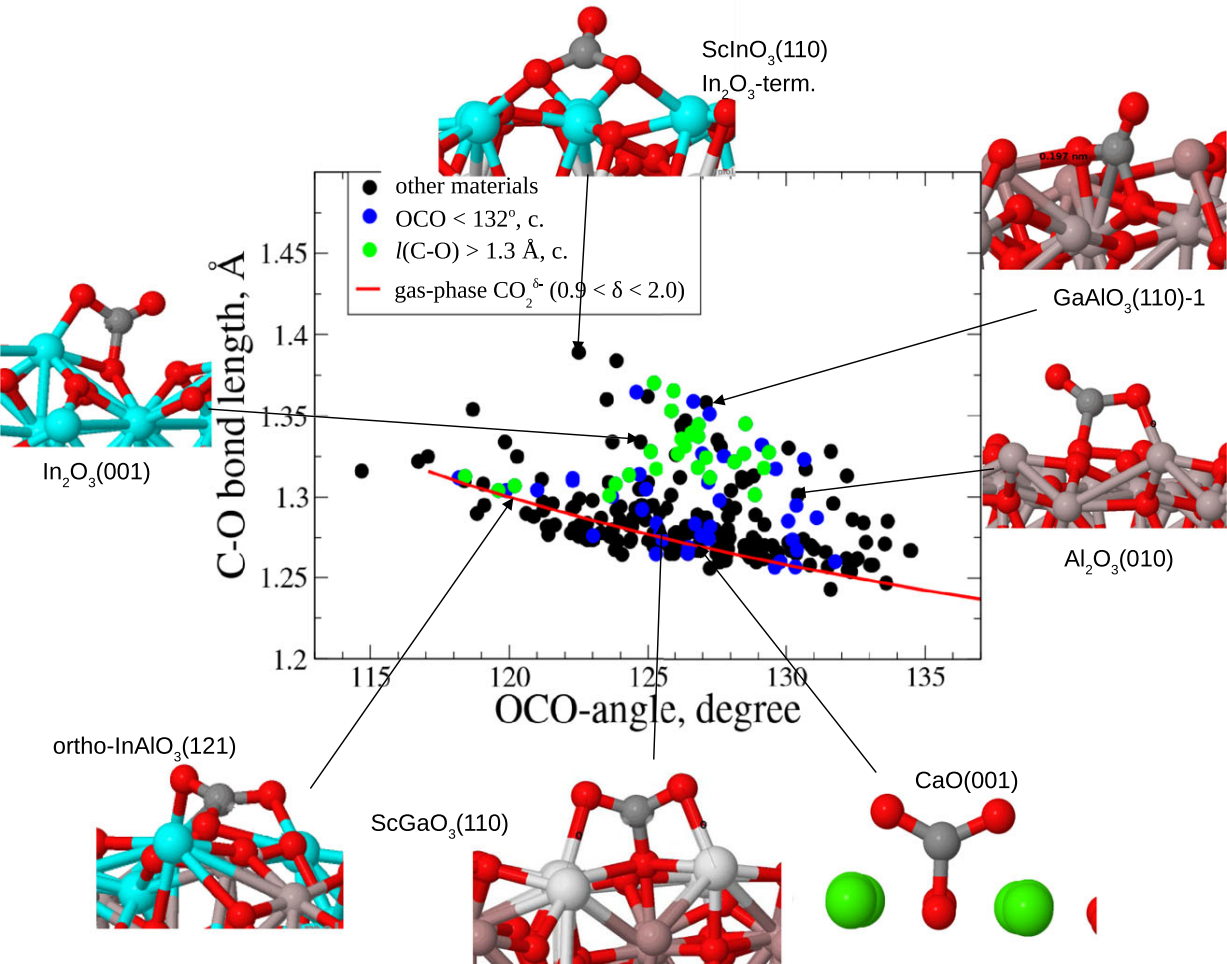
Correlation between the larger of the two C–O bond lengths and the OCO-angle for charged gas-phase and adsorbed CO_2_. The OCO-angle in charged gas-phase CO_2_ is shown with the red line, and adsorbed CO_2_ structures are shown with the dots. Colored dots: blue—adsorption sites from the unconstrained subgroup with OCO < 132°, green—subgroup of sites with *l*(C–O) > 1.30 Å, black—the remaining samples (see the text). The subgroups obtained with Sabatier principle constraint are marked with “c”.

We considered six different candidate indicators of CO_2_ activation, including OCO-angle and C–O bond distance. The bending of the OCO-angle in the adsorbed CO_2_ molecule relative to the gas-phase value of 180° (linear configuration) has been previously proposed^27^ and is widely accepted as a good indicator of activation. For gas-phase CO_2_, it is understood that the C–O double bond is weakened when an electron is added to the lowest unoccupied orbital, because it is of antibonding (π*) character with a concomitant bending of the molecule. There is a one-to-one mapping between the C–O bond length *l*(C–O) and the OCO-angle in gas-phase CO_2_^δ−^ for a range of δ > 0 (red curve in Fig. 1). However, this is not the case for the adsorbed CO_2_ (dots in Fig. 1). There is a subset of adsorbed CO_2_ that is close to the red line, but there are many cases where *l*(C–O) is substantially larger for a given OCO-angle. This is in contrast to metal alloy nanoparticle catalysts, where there is a better correlation between OCO-angle and *l*(C–O)^28^. Also, a longer C–O bond reflects a weakening and readiness for further chemical transformations. Thus, the bond elongation itself may be an alternative indicator of activation. A look at the adsorbed CO_2_ structures reveals that, on sites following the gas-phase correlation, the molecule adsorbs in nearly symmetric adsorption structures with nearly equal length of the two C–O bonds. In the other cases one O-atom of CO_2_ is close to surface cation(s), leading to a pronounced asymmetry of the adsorbed molecule.

Other considered potential indicators of activation include Hirshfeld charge^29^ of adsorbed CO_2_ (a direct indicator of the charge transferred to CO_2_), the dipole moment of the surface along the surface normal per adsorbed CO_2_ molecule (includes charge transfer to the molecule, as well as adsorption induced surface relaxation), the difference in Hirshfeld charges of C and O-atoms in an adsorbed CO_2_ molecule (indicates the ionicity of C–O bonds), and the difference in Hirshfeld charges of the O-atoms in the adsorbed molecule (indicates asymmetry of the adsorbed molecule)^9,29^.

### Subgroup discovery

To find out which properties (features) of the clean surfaces determine when a given activation indicator is maximized or minimized, we employ the subgroup-discovery (SGD) approach^30–34^. Given a dataset and a target property known for all data points, the SGD algorithm identifies subgroups with “outstanding characteristics” (see further for the criteria for being outstanding) and describes them by means of conjunction of basic propositions (selectors) of the kind “(*f*_1_ < *a*) AND (*f*_2_ ≥ *b*) AND ...”, where *f*_*i*_ is a feature and *a*, *b* are threshold values also found by SGD. In the framework of SGD, we call the selected primary features {*f*_1_, *f*_2_, ...} materials *genes*. Thus, SGD identifies both the outstanding subgroups and the relevant materials *genes* for a given target property.

Obviously, the selectors should only contain features that are much easier to evaluate than the target property. In the presented work, the considered features include properties of gas-phase atoms that build the material, and properties of the pristine material (properties of the bulk phase and of the pristine relaxed surface). Overall 46 primary features have been considered. The full list is presented Supplementary Table 3. Our strategy is to provide an almost exhaustive list of features, and use data analytics to select materials *genes* from this list. Some of these features have been explored previously as descriptors of catalytic activity for semiconducting and metallic oxides^35–38^. O 2*p-*band center features have been shown to correlate with catalytic properties of both semiconducting and metallic oxides^35,37^. In particular, most of the features (or closely related ones) mentioned in ref. ^36^, inspired by the work of Grasselli^39^, are included in our set, except oxygen vacancy formation energy, which is relevant for the oxidation catalysis, while here we are interested in partial or complete reduction. Additional important features in our work (see below) include features related to the polarizability of surface cations, which describe the long-range surface response to charged adsorbates. A subset of features from our list has been recently used successfully for predicting catalytic properties of metallic oxides^38^, along with additional features relevant specifically for metallic oxides (such as partial electronic state fillings).

The features selected by the SGD are summarized in Table 1.

**Table 1:** Features that appear in the top SGD selectors (see text).

symbol	Meaning
*IP*_min/max_	Ionization potential, minimal and maximal in the pair of atoms *A* and *B*; calculated as *E*_atom_ − *E*_cation_
*EA*_min*/*max_	Electron affinity, minimal and maximal in the pair of atoms *A* and *B*; calculated as *E*_anion_ − *E*_atom_
*EN*_min/max_	Mulliken electronegativity, minimal and maximal in the pair of gas-phase atoms *A* and *B*
*r*_−1_^min^, *r*_−1_^max^	Radii of the maximum value of the Kohn-Sham radial wave functions of the spin-unpolarized spherically symmetric atom for HOMO-1, maximum (max) and minimum (min) in the pair of atoms *A* and *B*
*r*_+1_^*min*^*, r*_+1_^*max*^	Radii of the maximum value of the Kohn-Sham radial wave functions of the spin-unpolarized spherically symmetric atom for LUMO, maximum (max) and minimum (min) in the pair of atoms *A* and *B*
*M*	Energy at which the surface O 2*p*-band projected density of states (PDOS) is maximal
*d*_1_, *d*_2_, *d*_3_	Distances from surface O-atom to the first-, second-, and third-nearest cations
*W*	Work function *W*, as the negative of the valence-band maximum (*W* = −*VBM*) with respect to vacuum level
*q*_min_, *q*_max_	Minimal and maximal Hirshfeld charges of cations in the pair *A* and *B*, calculated as an average for all surface cations of a given type
Δ	Bandgap
*CBM*	Conduction band minimum
*Q*_5_, *Q*_6_	Local-order parameter with *l* = 5 or 6
*PC*	Weighted surface O 2*p*-band center
*α*_O_, *C*_6_^O^	Polarizability and *C*_6_-coefficient for surface O-atom obtained from many-body dispersion scheme
α_min_, α_max_, *C*_6_^min^, *C*_6_^max^	Polarizability and *C*_6_-coefficient for cations, minimal and maximal in the pair *A* and *B*, calculated as an average for all surface cations of a given type
*q*_O_	Hirshfeld charge of O-atom at the surface
*wid*	Square root of the second moment of surface O 2*p*-band
*wid*_min_, *wid*_maxS_	Square root of the second moment of PDOS of cations within valence-band, minimal and maximal in the pair *A* and *B*, calculated as an average for all surface cations of a given type
*c*_min_, *c*_max_	First moment for PDOS of cation within valence-band, minimal and maximal in the pair *A* and *B*, calculated as an average for all surface cations of a given type
φ_1.4_, φ_2.6_, φ_1.4_ - φ_2.6_	Electrostatic potentials above surface O-atom at 1.4 and 2.6 Å and their difference. 1.4 Å corresponds to the average length of the bond between C and surface O, 2.6 Å is the minimal distance from surface O to C-atom of physisorbed carbon-dioxide molecule as observed from our calculations
*L*_min_, *L*_max_	Energy of lowest unoccupied projected eigenstate of surface cations, minimal and maximal in the pair *A* and *B*, calculated as an average for all surface cations of a given type
*kurt*	Kurtosis of surface O 2*p*-band PDOS
*U*	Eigenstate with least negative value in surface O 2*p*-band
*BV*	Bond-valence value of surface O-atom

The outstanding subgroup should satisfy several criteria. It should be statistically relevant; therefore the subgroups of too small size should be penalized. Target-property values (OCO-angle, C–O bond length, etc.) for subgroup samples should be as different as possible from corresponding gas-phase values since their change upon adsorption indicates CO_2_ activation^33^. To achieve this, two requirements are imposed simultaneously: (i) The target-property values for subgroup members should be smaller or larger (depending on the target) than a certain value (a cutoff), and (ii) the target-property values are minimized or maximized within the cutoff. The latter condition gives preference to subgroups with smaller or larger target-property values among similarly sized subgroups within the cutoff. The value of the cutoff is a parameter. As it approaches the optimal value of an activation indicator among all data points, additional or alternative materials *genes* and their combinations leading to stronger activation are identified. We explore the whole range of the parameter for each target property (for OCO-angle—123°, 124°, 126°, 128°, 130°, and 132°; for *l*(C–O)—1.26 Å, 1.28 Å, and 1.30 Å).

In addition to these criteria, we consider the requirement that adsorption energies are not too strong and not too weak for most of the samples in a subgroup. Strong activation (i.e., strong weakening of the C–O bonds) can be achieved by strong binding to the surface. It is well known that good catalytic performance requires a balanced adsorption strength. This is known as Sabatier principle. In addition to the practical value of identifying subgroups that satisfy this principle, comparison of subgroup selectors obtained with and without this requirement helps to identify combinations of materials features that promote desired changes in target properties and at the same time yield intermediate adsorption energies.

Sabatier principle is reflected by a characteristic volcano-type behavior of catalytic activity as a function of adsorption energy of reactants and intermediates. The position of the top of the volcano depends on particular reactions and conditions. It can be estimated from condition |Δ*G*| ~ 0, where Δ*G* is the Gibbs free energy of adsorption. For CO_2_ adsorption at room temperature and partial CO_2_ pressure of 1 atm this condition corresponds to about −0.5 eV adsorption energy^40^. At temperatures around 450 °C (typical conditions for CO_2_ methanation^41^) Δ*G* = 0 corresponds to adsorption energy −1.7 eV^41^. Therefore, for catalytic conversion at low or moderate temperatures this implies that CO_2_ adsorption energies should be in the range from between −2.0 and −0.5 eV.

These requirements are implemented in the following quality functions that are maximized during the search for subgroups. In particular, for OCO-angle minimization we use:

1 $$F(Z)={\theta }_{{{{{{\mathrm{cut}}}}}}}\left[\frac{s(Z)}{s(Y)}\cdot \left(\frac{{{\max }}(Z)-{\alpha }_{g}}{{{\min }}(Y)-{\alpha }_{g}}\right)\cdot u(p)\right]$$

and for C–O bond maximization the following quality function was applied:

2 $$F(Z)={\theta }_{{{{{{\mathrm{cut}}}}}}}\left[\frac{s(Z)}{s(Y)}\cdot \left(\frac{{{\min }}(Z)-{l}_{g}}{{{\max }}(Y)-{l}_{g}}\right)\cdot u(p)\right]$$

where *Y* is the whole dataset, *Z*—a subgroup, *s*—size (number of data points), min and max – minimal or maximal value of the target property, *α*_g_ and *l*_g_ are the gas-phase values of OCO-angle and C–O bond distance, 180° and 1.17 Å, respectively, and *θ*_cut_ is the Heaviside step function which is equal 1 if all data points in the subgroup satisfy the cutoff condition and 0 otherwise. Thus, larger values of the quality function *F*(*Z*) are obtained for those subgroups in which minimal (maximal) value of a target property is close to the maximal (minimal) value of the whole sampling with respect to the gas-phase value of CO_2_ molecule. The use of maximum/minimum instead of a median is done to ensure that a target property is optimal for as many members of a subgroup as possible. The gas-phase reference values are usually significantly different from the “chemisorption” subset. Therefore, the term in squared brackets in Eqs. (1) and (2) can noticeably contribute only when the sizes of candidate subgroups are similar.

The term *u*(*p*) in Eqs. (1) and (2) is added in order to account for Sabatier principle in SGD framework. We have implemented a multitask quality function, where a factor *u*(*p*) increases the quality of subgroups with adsorption energies falling within this range. This is formulated in terms of the information gain^34^, i.e., reduction of the normalized Shannon entropy. We perform the SGD for each target property both explicitly accounting for the Sabatier principle and without it. The latter case is equal to *u*(*p*) = 1 in Eqs. (1) and (2)^34^.

We note that SGD is qualitatively different from machine-learning classification/regression techniques such as neural networks, kernel regression methods, or decision-tree regression (DTR^42^) (e.g., random forest). SGD is typically referred to as a supervised descriptive rule-induction technique^43^, i.e., it uses the labels assigned to the data points (the values of the target property) in order to identify patterns in the data distribution (the statistically exceptional data groups) and the rules defining them (the selectors), by optimizing a quality function which is a function of the distribution of values of the target property^43^. While there are apparent similarities between SGD and DTR as both methods yield models in terms of physically interpretable selectors (usually, inequalities) on a selected subset of the input features, the analogy stops at this level, as SGD focuses at (and only at) subgroups from the very beginning and says nothing about the data that are not in the subgroup. In contrast, DTR determines a global partitioning of the input space by minimizing a global quality function, i.e., the quality of a single subset is secondary with respect to the resulting quality of all subsets partitioning the whole dataset. In other words, for finding distinct combinations of materials genes driving desirable changes in a particular target property (possibly different combinations leading to the same result), the SGD approach has significantly higher flexibility and reliability. This is demonstrated below for a DTR analysis for our target properties.

The metadata and workflow for the AI analysis are documented in the Jupyter notebook^26^.

### Results of the subgroup discovery

The SGD for OCO-angles was done with Eq. (1) for the quality function, and OCO as a target property, since smaller angles indicate larger charge transferred to the molecular π^*^ orbital. The subgroup selectors obtained with different OCO-angle cutoffs (126°, 128°, 130°, and 132°) with or without the adsorption energy constraint are listed in Table 2 (for more details see the Supplementary Table 4). Analysis of these subgroups reveals that the angle reduction is determined by an interplay of several factors: an electron transfer from the cations to surface O-atoms, delocalization of electron density between cations and O-atoms, and coordination of the surface O-atoms. Without the Sabatier principle constraint, the OCO-angle reduction below 132° is mainly due to the electron accumulation at the O-atom of the clean surface. This is expressed by the conditions of more negative Hirshfeld charge on O-atoms (*q*_O_ < …), not very low IP of at least one cation (*IP*_max_ > …), and increased polarizability of the surface O-atom on which CO_2_ is adsorbed (*C*_6_^O^ > ...). Upon adsorption of CO_2_, this charge on the surface O-atom is readily available for transfer to CO_2_. When the Sabatier principle constraint is introduced, the OCO < 132° subgroup also includes sites with a pronounced electron transfer to CO_2_, but with a lower-energy O 2*p-*band maximum (*M* < ...) with respect to vacuum level, and a larger kurtosis (*kurt* > ...). These conditions imply reduced inter-electronic repulsion around the surface O-atom achieved by partial delocalization of the charge density.

**Table 2:** Top subgroups and their selectors obtained by minimization of OCO-angle and maximization of *l*(C–O) with/out Sabatier principle (energies are in eV, distances are in Å, charges are in units of absolute electron charge, polarizabilities are in Bohr^3^).

cutoff	size	selector	cutoff	size	selector
OCO minimization without Sabatier principle constraint			OCO minimization with Sabatier principle constraint		
126	19	*L*_max_ > −2.70 (*L*_min_ > −2.19, *CBM* > −3.40, *r*_+1_^max^ ≤ 2.83, W < 5.80, U > −5.61) *IP*_max_ ≥ −6.05 α_max_ ≤ 184.5 Δφ > 1.33 *q*_max_ ≤ 0.59 *wid* ≤ 1.59 *wid* ≥ 0.58	126	15	*L*_*min*_ ≥ −5.1085 φ_*2.6*_ ≤ 0.3033 Δφ ≤ 1.0622 (*c*_max_ ≤ −8.5915) *d*_*1*_ ≥ 1.82 *d*_*2*_ ≥ 2.005 *r*_+1_^max^ > 2.83
128	44	*EA*_max_ ≥ −0.43 *Q*_6_ ≥ 0.51 α_max_ ≥ 50.4 (*C*_6_^max^ ≥ 389.5, α_O_ ≤ 2.70) Δφ ≥ 1.00 *q*_min_ ≤ 0.49	128	30	*C*_6_^min^ ≥ 369.5 *L*_max_ ≥ −4.73 (*r*_+1_^min^ ≤ 2.82, *IP*_min_ ≤ −5.83, *r*_HOMO_^min^ ≤ 1.41) Q_5_ ≤ 0.83 Δφ ≥ 0.60 *r*_+1_^max^ ≥ 2.80 *C*_6_^O^ ≤ 12.10
130	77	*L*_max_ ≥ −5.23 *EA*_max_ ≤ 0.16 (*C*_6_^max^ ≥ 389.5, *IP*_max_ ≥ −7.00) *d*_1_ ≥ 1.82 *d*_2_ > 2.10	130	40	φ_2.6_ ≥ −0.15 Δφ ≥ 0.73 *d*_1_ ≤ 2.01 *d*_2_ ≥ 1.96 *d*_3_ ≥ 2.025 (*c*_min_ ≤ −9.07, *W* ≥ 5.10) *q*_min_ ≤ 0.49 *r*_+1_^min^ ≥ 1.94
132	139	*IP*_max_ ≥ −6.99 *q*_O_ ≤ -0.32 *C*_6_^O^ ≥ 10.36	132	58	*q*_O_ ≤ −0.3386 *M* ≤ −6.292 *kurt* ≥ 2.1035 *IP*_max_ ≥ −6.2085 *r*_HOMO_^min^ ≤ 1.407 (*IP*_min_ ≤ −5.91, *r*_+1_^min^ ≤ 2.82)
*l*(C–O) maximization without Sabatier principle constraint			*l*(C–O) maximization with Sabatier principle constraint		
1.26	121	*C*_6_^min^ ≥ 343.5 φ_2.6_ ≤ 0.66 *Q*_5_ ≤ 0.83 *M* ≥ −8.05 (*PC* ≥ −9.32)	1.26	56	*CBM* ≥ −5.17 (*L*_min_ ≥ −5.11) Δφ ≤ 1.13 *PC* ≥ −8.62 *d*_3_ ≤ 2.48 *M* ≤ −6.06
1.28	38	*EA*_max_ ≤ 0.005 *d*_2_ > 2.22 *M* ≤ −4.12	1.28	30	*W* ≥ 5.10 (*M* ≤ −5.19, *U* ≤ -4.92, *PC* ≤ −7.21) *d*_2_ > 2.14 *q*_min_ < 0.48
1.30	27	*U* ≤ −5.34 *d*_2_ > 2.14 *q*_min_ < 0.48 *kurt* ≥ 2.10 (*q*_max_ ≥ 0.47)	1.30	27	*EA*_max_ ≤ 0.005 (*W* ≥ 5.10, *M* ≤ −5.19, *U* ≤ −4.92, *PC* ≤ −7.21) *EN*_min_ ≤ −3.19 (*W* ≥ 5.10, q_O_ ≥ −0.45, *c*_max_ ≤ −7.18, *r*_HOMO_^min^ ≤ 1.41, φ_1.4_ ≤ 2.40, *c*_min_ ≤ −8.135, *q*_max_ ≥ 0.47, *M* ≤ −5.19, *IP*_min_ ≤ −5.91, *wid* ≥ 0.58, *U* ≤ −4.92, *r*_−1_^max^ ≥ 0.97, *PC* ≤ −7.21, Δφ ≤ 1.81) *d*_2_ > 2.14 *q*_min_ < 0.48 *kurt* ≥ 2.51

Proposition replacements that do not change the support are shown in parentheses.

At lower OCO cutoffs, the subgroup selectors include coordination descriptors *Q*_*i*_, *i* = 5, 6. Without Sabatier principle, sites with larger *Q*_*i*_ are selected, and vice versa. Larger *Q*_*i*_ indicates lower coordination of the O-atom. This reduces electron repulsion and therefore facilitates electron transfer to the O-atom of the clean surface. However, this also increases the bonding strength of CO_2_ to the surface. This explains why selectors of subgroups obtained with Sabatier principle include the opposite conditions (*Q*_*5*_ < ...).

Other surface features describing electron distribution are related to Madelung potential: electrostatic potential and field (φ_1.4_, φ_2.6_, and Δφ = φ_1.4_ − φ_2.6_) and distances between the O-atom and surface cations. More open surface structure with larger distances between cations at the O site facilitates charge transfer to adsorbed CO_2_ molecule, since the Madelung potential from the nearby cations is reduced. This is reflected in the appearance of propositions involving features *d*_1_, *d*_2_, and *d*_3_. For example, for the OCO ≤ 130° subgroups, imposing energy constraint changes proposition (*d*_1_ > ...) to (*d*_1_ < ...), which implies an increased energy cost for transferring electrons to CO_2_. Larger electric fields Δφ around the adsorption site imply stronger localization of electron density on O-atoms, and thus also improve the efficiency of charge transfer to the adsorbed molecule.

The smaller OCO subgroups with Sabatier principle also include propositions implying increased polarizability of both cations (*C*_6_^min^ > ...). Another support-defining condition is that the radius of the lowest unoccupied orbital for the metal atoms should not be small (*r*_+1_ ≥ ...). This requirement is true for most cations with negative electron affinities (Supplementary Fig. 4). Analysis of adsorbed CO_2_ structures and Hirshfeld charges reveals that this condition together with the higher polarizability of cations at the *pristine* surface encompasses two scenarios: (i) additional electron transfer to CO_2_ upon adsorption and (ii) stronger binding between O-atoms in CO_2_ and surface cations. When scenario (ii) dominates, CO_3_^δ−^ anion lies nearly horizontally at the surface, and is bound with nearby cations by chemical bonds via its oxygen atoms. Such a structure leads to small OCO-angles in CO_3_^δ−^ (around 120°), even if charge transfer is limited. Thus, increased bending of adsorbed CO_2_ occurs due to charge transfer over larger distances and/or distortion of the adsorbed molecule and the surface, both leading to weaker adsorption. The cases where both scenarios are active include the same sites as in the subgroups with elongated *l*(C–O), as described below.

In order to obtain the subgroups of adsorption sites with larger *l*(C–O), we performed the SGD with the quality function Eq. (2) and *l*(C–O) as target property. The results for *l*(C–O) cutoffs 1.26, 1.28, and 1.30 Å are summarized in Table 2 and Supplementary Table 5. In contrast to OCO, the analysis of the obtained top subgroups shows a much less pronounced or no effect of imposing Sabatier principle on the distribution of adsorption energies within the subgroups. This is because sites with too strong adsorption are excluded based on *l*(C–O) threshold alone, without the need to introduce the energy constraint. For example, the range of *l*(C–O) for the top *l*(C–O) > 1.26 Å subgroup without constraining adsorption energies is the same as for the top OCO < 130° subgroup, but it contains significantly more sites with intermediate adsorption energies.

Electron transfer to an adsorbed CO_2_ molecule increases both the OCO bending and C–O bond elongation. The main difference between OCO and *l*(C–O) subgroups is that in the latter an additional mechanism of increasing *l*(C–O) is in effect, namely a covalent bonding between one O-atom of the CO_2_ molecule and the nearest surface cation. This can be concluded from the analysis of adsorption geometries, and correlates with the presence of proposition (*EA*_max_ ≤ 0.005 eV), selecting cation species that can accept electron density, e.g., from an O-atom in adsorbed CO_2_ molecule. Other proposition that appears in most selectors of top subgroups is (*d*_2_ > 2.14 Å) or (*d*_2_ > 2.22 Å)—larger distances to the second nearest cation from an O-atom. Larger elongation of the C–O bond is achieved by the asymmetry of the cation types at the surface, where one can bind an O-atom of the adsorbed CO_2_, while the other (located further away) cannot. An example asymmetric CO_2_ adsorption structure is shown in Supplementary Fig. 5.

Other propositions indicate a moderate charge transfer to adsorbed CO_2_ molecule as in the case of OCO subgroups with adsorption energy constraint. Propositions (*M* ≥ −8.05 eV), (*PC* ≥ −9.32 eV) in *l*(C–O) < 1.26 Å subgroups imply enhanced charge density on the surface O-atoms, since electron–electron repulsion raises energies of O 2*p-*band states. However, at larger *l*(C–O) cutoffs the electron transfer is balanced by such propositions as (*M* ≤ −5.19 eV), (*U* ≤ −4.92 eV), and (*W* ≥ 5.10 eV) indicating limited electron transfer. These propositions point to more covalent bonding between cations and surface O-atom. Rather persistent proposition observed in many selectors of *l*(C–O) subgroups is the limit of minimal charge on surface cations (*q*_min_ < 0.48*e*). It also shows the limitation of the charge transfer from one type of cations to surface oxygen atoms.

In general, we find that subgroups obtained with smaller cutoffs do not have a strong overlap with subgroups with larger cutoffs for OCO. In particular, for subgroups with close cutoffs the overlap can be smaller than 50% of the smaller subgroup (but is never below 30%). Interestingly, for *l*(C–O) the situation is opposite: subgroups with tighter cutoffs are mostly contained in the subgroups for more relaxed constraints. This means that, while larger values of *l*(C–O) are mainly controlled by the same or additional *genes*, smaller values of OCO are due to alternative *genes*. The overlap of OCO subgroups becomes even smaller when Sabatier principle is included, confirming the absence of a universal mechanism for OCO-angle reduction that is compatible with moderate adsorption energy.

**Fig. 2:**
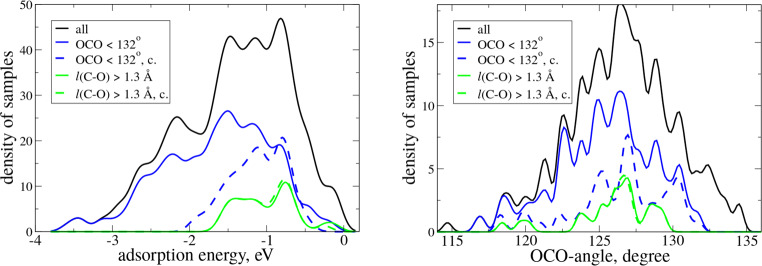
Distribution of adsorption energies (left) and OCO-angles (right). The distribution is shown for the whole dataset (black), for the top subgroups of sites with OCO < 132° angles (blue) and *l*(C–O) > 1.30 Å (green). The subgroups obtained with adsorption energy constraint are marked with “c.” and shown with dashed lines. The adsorption energy *E*_ads_ is defined as the difference between the total energy of the slab with adsorbed CO_2_ and the sum of total energies of the clean slab and an isolated CO_2_ molecule.

In summary, we find that, while an increased electron density at the O adsorption site is necessary for chemisorption and leads to both OCO bending and C–O bond elongation in an adsorbed CO_2_ molecule, there are additional actuators for these effects that are different for different target properties. The OCO-angle is in general minimized by increasing electron transfer to the O site. However, this also leads to strong adsorption for many materials (Fig. 2). To satisfy Sabatier principle, the electron transfer to CO_2_ must be moderate. This is achieved by delocalization of charge density around O sites and/or by distortion of the adsorbed molecule due to the formation of covalent bonds between O-atoms in CO_2_ and surface cations. The largest C–O bond elongations are achieved when both charge transfer to adsorbed CO_2_ and the covalent interaction are present, and local geometry around surface O-atom provides the asymmetry in adsorption structure. This mechanism automatically fulfills the Sabatier principle.

The subgroups found by SGD for the dipole moment induced by CO_2_ adsorption, its total Hirshfeld charge, and the difference of charges on C and O-atoms significantly overlap with the subgroup of smaller OCO-angles. The subgroup found by maximizing the difference of Hirshfeld charges on O-atoms of an adsorbed CO_2_ largely overlaps with the subgroup of sites delivering larger *l*(C–O). In general, these indicators are not better than OCO or *l*(C–O). Therefore, below we focus on OCO-angle and *l*(C–O) as indicators of CO_2_ activation. More details about the other indicators can be found in Supplementary Discussion.

### Comparison with experimental results

To address the question which of the discussed properties can serve as an indicator of the catalytic activity, we compare our predictions to reported experimental results (Table 3). It should be stressed that the available experimental data are scarce, and results are difficult to compare quantitatively. We consider thermally and, for completeness, some photo-driven catalysis and thus also include supported metal catalysts with the considered oxides as support. Despite possibly different mechanisms for CO_2_ conversion in the different types of catalysis, we believe that the properties of adsorbed CO_2_ molecule can still serve as indicators of catalytic activity. Thus, it is possible that under such a daunting situation a reliable indicator of CO_2_ activation can still be identified. As described below, our analysis confirms this hope.

**Table 3:** The catalytic performance of materials which contain the sites from larger *l*(C–O)) or/and smaller OCO subgroups.

Material	Catalytic reaction	CO_2_ adsorption energies, eV	Belong to subgroups
NaNbO_3_	Photocatalytic CO_2_ reduction with ~70% of CO selectivity^46, 48^	−0.77 to −0.81	Materials with sites from *l*(C–O) > 1.30 Å subgroup and OCO < 132° subgroup with Sabatier principle constraint
LaAlO_3_	Dry reforming of methane with Ni-nanoparticles; performance is higher than for Ni-La_2_O_3_ and Ni-Al_2_O_3_^45^	−1.17	
KNbO_3_	Photocatalytic reduction of CO_2_ into CH_4_ as a composite with Pt/g-C_3_N_4_; significant improvement of activity when compared to Pt/g-C_3_N_4_; Pt-KNbO_3_ is ~2.5 times more photoactive than Pt-NaNbO_3_^46, 47^	−0.56 to −0.68	
CaTiO_3_	CO_2_ hydrogenation under UV-irradiation, although activity is not very high^51, 57^; twice higher activity with Ni-nanoparticles^57^	up to −2.70	Materials with sites from *l*(C–O) > 1.30 Å subgroups and from OCO < 132° subgroup without Sabatier principle constraint
CaZrO_3_, SrZrO_3_, BaZrO_3_, SrTiO_3_	Reverse water gas-shift reaction (RWGS) under 700–1100 °C^49^	up to −2.75	
SrTiO_3_	Photocatalytic CO_2_ methanation with Pt, Au-nanoparticles, significant decrease of activity during reaction^50^	up to −2.40	
YInO_3_^a^	No activity observed in photocatalytic CO_2_ conversion^52^	−1.16–−1.47	Materials with sites only from OCO < 132° subgroup without Sabatier principle constraint
CaO, SrO, BaO, Na_2_O	Strong carbonation, candidate materials for carbon capture and storage (CCS)^44^	−1.60 to −3.57	
La_2_O_3_	Dry reforming of methane with supported Ni-nanoparticles; lower performance than on Ni-LaAlO_3_^45^ and on some other supported catalysts^54^ at 700 and 250 °C correspondingly	−2.14 to −3.11	
CaO	Twice smaller reaction rate in CO_2_ reforming of methane reaction with supported Ni-nanoparticles than on Ni-La_2_O_3_^58^ at 750 °C	−1.60 to −3.42	
Ga_2_O_3_	Electrochemical reduction of CO_2_ to formic acid^59^; (photo)catalytic hydrogenation of CO_2_^60^	−0.74 to −1.34	Materials with sites from OCO < 132° subgroup with Sabatier principle constraint
Al_2_O_3_	Dry reforming of methane with supported Ni-nanoparticles^61^; lower performance than on Ni-LaAlO_3_^45^	−0.87	

^a^Materials with sites also from OCO < 132° subgroup with Sabatier principle constraint.

First, we consider materials with the sites from subgroups obtained by minimization of OCO-angle without Sabatier principle constraint^27^. For quite many materials from these subgroups, independent of the cutoff value, there are no reports of successful CO_2_ conversion, even when they are used as supports for metal nanoparticles (Table 3). This is explained by the fact that absolute adsorption energies for these materials are above 2 eV (Fig. 2 left, Supplementary Table 4), indicating that their surfaces will be permanently poisoned by carbonate species at low or intermediate temperatures. This means that on materials with these sites hardly any reaction of CO_2_ conversion can proceed at low, especially room temperature. Moreover, as shown in Table 3, even at increased temperatures, 700–750 °C, the activity of these materials is low. Some of them have been considered as candidates for carbon capture and storage (CaO, SrO, BaO, and Na_2_O)^44^, which implies the formation of stable carbonates rather than CO_2_ transformation. Thus, we conclude that OCO-angle alone is not a good indicator of enhanced catalytic activity in CO_2_ conversion.

On the other hand, several of the materials with sites from *l*(C–O) > 1.30 Å subgroups (independent on either with or without Sabatier principle constraint) are known as good materials for CO_2_ conversion (Table 3) in different reactions proceeding at room or higher temperatures. For these sites, the absolute adsorption energies already satisfy the Sabatier principle (Fig. 2, left), as discussed above. We note that, contrary to what one may expect, there is no correlation between the adsorption energy and the value of *l*(C–O) (see Supplementary Fig. 5). Although there is a general trend, there are also significant variations in *l*(C–O) for given adsorption energy.

Interestingly, some of the materials with sites in the *l*(C–O) > 1.30 Å subgroups were studied as supports for metallic nanoparticles. For instance, Ni/LaAlO_3_ is a catalyst for dry reforming of methane^45^ at 700 °C. It was shown that its catalytic performance is higher in terms of CO_2_ and CH_4_ conversion rates compared to Ni/La_2_O_3_ and Ni/Al_2_O_3_^45^. All sites on considered lanthanum (III) oxide surfaces belong to the subgroup of OCO < 132° without Sabatier constraint, whereas the sites on Al_2_O_3_ do not enter any of the two subgroups. KNbO_3_ has been studied only with Pt nanoparticles and as a composite with g-C_3_N_4_ in photocatalytic reduction of CO_2_ into CH_4_^46,47^. Pt-KNbO_3_ is ~2.5 times more photoactive than Pt-NaNbO_3_^46^, whereas the NaNbO_3_ is known to be photoactive even without nanoparticles^48^. This seems to suggest that *l*(C–O) is a good indicator of CO_2_ activation for both unsupported and supported catalysts even at increased temperatures. Hence, the other materials with the sites from this subgroup are promising new candidates for this task. The most promising materials identified in this work are CsNbO_3_, CsVO_3_, RbVO_3_, LaScO_3_, RbNbO_3_, and NaSbO_3_ as they have the sites from the larger *l*(C–O) subgroups satisfying the above-mentioned criteria.

There is also a set of materials [ternaries *A*^2+^*B*^4+^O_3_ (*A* = Ca, Sr, Ba, *B* = Zr, Ti, Ge, Sn, Si) with a perovskite structure] containing both the surfaces with sites from the smaller OCO subgroups without Sabatier constraint and the surfaces with sites from the larger *l*(C–O) subgroups (Table 3). These two types of sites are located on different surfaces. Thus, based on the above results, a material for which a surface with sites from the *l*(C–O) > 1.30 Å subgroups has lower formation energy and is more abundant than the surface with sites from smaller OCO subgroups without Sabatier constraint is expected to be a good catalyst. To explore this possibility, we analyze the surfaces of these materials in more detail. Their most stable surfaces are *A*O-terminated (001) facets containing sites from the smaller OCO subgroup. The formation energies of *AB*O_3_-terminated (110) surfaces with larger *l*(C–O) sites are higher: for BaZrO_3_, SrZrO_3_, CaZrO_3_, and SrTiO_3_ the differences in formation energies are 0.049, 0.027, 0.013, and 0.037 eV/Å^2^, respectively. The zirconates and SrTiO_3_ were found to catalyze the water gas-shift reaction under increased temperatures, 700–1100 °C^49^. At room temperature the photocatalytic activity of SrTiO_3_ was found to be significantly decreased^50^. We attribute the latter finding to the strong carbonation of its most stable surface, which is consistent with the calculated high absolute value of CO_2_ adsorption energy (−2.4 eV) for this surface. Thus, the activity of SrTiO_3_ at 700 °C and higher temperatures is consistent with the estimates of the CO_2_ chemical potential given above. The difference in formation energies of the most stable CaO-terminated (001) surface and the stoichiometric (110) surface for CaTiO_3_ is less pronounced compared to zirconates and other titanates (CaO-terminated (001) is more stable than the (110) surface by only 0.009 eV/Å^2^). Thus, the (110) facets, which contain sites from the long *l*(C–O) subgroup, may be present on catalyst particles at the reaction conditions. This can explain the observed activity of CaTiO_3_ in CO_2_ conversion not only at high but also at room temperature. We note that the activity of this material was also attributed to the presence of TiO_2_ nanoparticles on the surface^51^ at reaction conditions.

The OCO subgroup that includes most of the known good catalysts and a minimal number of inactive materials is OCO < 132° with Sabatier principle. It contains the sites on discussed above LaAlO_3_, KNbO3, and NaNbO_3_ catalysts, but also on non-active YInO_3_ according to ref. ^52^ (Table 3). This subgroup contains in addition the sites on a well-known CO_2_ conversion catalyst Ga_2_O_3_. We should mention that the catalytic activity of Ga_2_O_3_ has been attributed to its reducibility. According to Pan and coworkers^53^ CO_2_ molecules are activated via dissociation on surface O-vacancies. However, in ref. ^54^ only one Ga_2_O_3_ (100) surface was considered for which no energetically stable CO_2_ chemisorption structures were obtained with the PBE functional. We show in Supplementary Table 1 and Supplementary Fig. 1 that this functional underestimates CO_2_ adsorption energies. Moreover, in our study we considered also other surfaces and found stable CO_2_ chemisorption structures on these surfaces. Thus, activation of CO_2_ on Ga_2_O_3_ can indeed proceed on O-atoms as discussed in our study, even without surface O-vacancies. The subgroups with small OCO cutoffs, 123° and 124°, do not contain any sites on known active or non-active catalysts.

OCO < 132° subgroup with Sabatier principle contains a large number of sites with elongated C–O bonds. The overlap of this subgroup with *l*(C–O) > 1.30 Å subgroups is 19 samples (70% of the latter).

To demonstrate the advantages of SGD over DTR in finding materials *genes* and their optimal combinations, we have done a comparison of found SGD subgroups with DTR performance for *l*(C–O). DTR terminal nodes (leaves) with the largest average *l*(C–O) (Supplementary Figs. 2 and 3) include surface sites on materials prone to extremely strong carbonation (Table 2), and also sites at which CO_2_ prefers to physisorb, with *l*(C–O) = 1.17 Å. Also, one cannot check the effect of imposing the constraint as there is no standard way to mix regression and classification in DTR. Thus, DTR in contrast to SGD is not able to separate different activation modes and even fails sometimes in distinguishing activation from non-activation.

### Best materials for CO_2_ reduction among calculated ones

Now those good indicators of activation are identified (OCO with Sabatier principle and *l*(C–O)), all calculated materials can be ranked according to the value of these indicators (smaller OCO or larger *l*(C–O) indicate C–O bond weakening and therefore higher catalytic activity, provided adsorption energy is moderate). The resulting list of the most promising catalysts for CO_2_ conversion is presented in Table 4. Each surface is characterized by maximum *l*(C–O) and minimum OCO among all inequivalent sites on that surface. The materials with *l*(C–O) > 1.30 Å are listed in the order of decreasing *l*(C–O). Materials with OCO < 132° but *l*(C–O) < 1.30 Å are appended at the bottom of the list in the order of increasing OCO.

**Table 4:** Best materials and surface cuts for CO_2_ activation according to the *l*(C–O) and OCO indicators.

Material	Surface cut	*l*(C–O), Å	OCO, degree	*E*_ads_, eV	In l(C–O) > 1.30 Å subgroup	In OCO < 132° c. subgroup
According to *l*(C–O) indicator						
NaSbO_3_	100	1.370	125.21	−1.32	Yes	Yes
Ga_2_O_3_	212	1.365	124.57	−1.34		Yes
NaSbO_3_	010	1.365	125.95	−1.09	Yes	Yes
LiSbO_3_	010	1.359	126.66	−1.04		Yes
NaNbO_3_	100	1.353	125.87	−0.78	Yes	Yes
ScAlO_3_	010	1.351	127.25	−1.18		Yes
KSbO_3_	110	1.345	128.54	−0.72	Yes	Yes
LiNbO_3_	100	1.344	126.23	−0.87		
NaNbO_3_	010	1.344	126.85	−0.77	Yes	Yes
InScO_3_	121	1.342	126.26	−1.23		
CsNbO_3_	100	1.34	126.6	−0.87	Yes	
RbNbO_3_	111	1.338	126.61	−1.37	Yes	Yes
CsNbO_3_	010	1.336	126.23	−1.11	Yes	
MgSnO_3_	100	1.334	119.84	−1.58		
GaAlO_3_	100	1.332	129.12	−1.02		Yes
CaGeO_3_	001(GeO_2_-term.)	1.331	127.65	−0.75		
InAlO_3_-or.	121	1.33	130.09	−1.02		
ScAlO_3_	121	1.328	131.61	−0.86		
GaInO_3_	110	1.327	126.98	−1.34	Yes	
LaAlO_3_	110	1.327	129.38	−1.17	Yes	Yes
CsVO_3_	110	1.327	126.1	−0.72	Yes	
KNbO_3_	110	1.327	128.49	−0.68	Yes	Yes
RbVO_3_	110	1.326	126.04	−1.14		
Ga_2_O_3_	110	1.325	127.76	−1.09		Yes
NaVO_3_	110	1.324	127.12	−0.755	Yes	
NaNbO_3_	110	1.322	128.14	−0.805	Yes	Yes
InAlO_3_-rh.	110	1.318	126.83	−0.73	Yes	Yes
LaGaO_3_	100	1.317	125.29	−0.97	Yes	
ScGaO_3_	010	1.314	124.68	−1.06		Yes
GaInO_3_	120	1.313	118.41	−1.43	Yes	Yes
MgGeO_3_-tetr.	001(GeO_2_-term.)	1.312	126.18	−1.35		
ScAlO_3_	100	1.312	122.28	−1.89		Yes
YAlO_3_	011	1.312	127.26	−1.18	Yes	Yes
InScO_3_	110	1.31	122.28	−1.54		Yes
In_2_O_3_	111	1.309	128.44	−0.65		
InAlO_3_-or.	110	1.309	127.2	−0.66		Yes
YAlO_3_	100	1.308	123.82	−1.305	Yes	Yes
InScO_3_	110(In_2_O_3_-term.)	1.305	124.92	−1.57		Yes
YGaO_3_	100	1.305	124.76	−1.23		
In_2_O_3_	110	1.301	125.86	−1.00		
Sc_2_O_3_	111	1.301	130.43	−0.885		
LaGaO_3_	110	1.301	128.88	−0.83	Yes	Yes
LaScO_3_	100	1.301	123.6	−1.53	Yes	
according to OCO indicator						
CaSiO_3_	001(CaO-term.)	1.290	118.84	−1.54		
SrSiO_3_	001(SrO-term.)	1.295	119.10	−1.66		
CaGeO_3_	001(CaO-term.)	1.288	120.88	−1.94		
Ga_2_O_3_	212	1.297	121.21	−1.53		
InScO_3_	110	1.292	121.23	−1.88		
InScO_3_	100	1.277	121.40	−1.74		
RbVO_3_	100	1.283	121.64	−0.53		
In_2_O_3_	110	1.280	122.52	−1.57		
InScO_3_	110(In_2_O_3_-term.)	1.284	122.80	−1.78		
SrGeO_3_	100(SrO-term.)	1.277	122.90	−1.70		
TiO_2_-rutile	100	1.276	123.61	−1.05		
ZrO_2_	111	1.280	123.72	−0.92		
BaSnO_3_	001(BaO-term.)	1.267	123.80	−1.89		
ScGaO_3_	110	1.292	123.85	−1.22		
ZrO_2_	011	1.264	124.06	−0.72		
LiVO_3_	110	1.295	124.76	−0.70		
NaNbO_3_	010	1.273	125.00	−1.66		
MgTiO_3_	012	1.295	125.16	−1.47		
InAlO_3_-or.	010	1.284	125.30	−0.82		Yes
YInO_3_	100	1.293	125.69	−1.47		
KNbO_3_	010	1.277	125.97	−1.52		
InAlO_3_-or.	110	1.278	126.04	−0.90		
ScAlO_3_	110	1.277	126.10	−1.33		
Al_2_O_3_	012	1.265	126.46	−0.87		Yes
Sc_2_O_3_	110	1.265	126.47	−1.14		
CaSiO_3_	110(CaO-term.)	1.278	126.49	−1.44		
LaInO_3_	100	1.287	127.13	−1.27		
Sc_2_O_3_	111	1.265	127.49	−0.95		
YInO_3_	110	1.298	127.61	−1.22		Yes
ScAlO_3_	121	1.268	127.73	−0.755		
MgTiO_3_	001	1.265	127.85	−1.37		
BaGeO_3_	001(BaO-term.)	1.270	128.50	−1.80		
SrTiO_3_	001(TiO_2_-term.)	1.266	128.53	−1.92		
ZnO	10–10	1.270	128.60	−1.005		
YGaO_3_	110	1.263	128.68	−1.60		
SrSnO_3_	001(SnO_2_-term.)	1.273	128.90	−1.64		
Sc_2_O_3_	001	1.289	128.90	−1.70		
MgGeO_3_	001	1.260	128.93	−1.09		
CaO	001	1.262	129.20	−1.60		
Al_2_O_3_	001	1.283	129.22	−1.315		
BaSnO_3_	001(SnO_2_-term.)	1.270	129.50	−1.87		
CaSnO_3_	001(SnO_2_-term.)	1.272	130.09	−1.32		
KVO_3_	010	1.267	130.17	−0.55		
CaZrO_3_	101(ZrO_2_-term.)	1.265	130.36	−1.86		
CaSnO_3_	110(SnO_2_-term.)	1.272	130.50	−1.44		
SrGeO_3_	100(GeO_2_-term.)	1.270	130.90	−1.515		
CaTiO_3_	101(TiO_2_-term.)	1.266	131.42	−1.505		
SnO_2_	100	1.257	131.50	−0.85		
BaSiO_3_	100	1.243	131.60	−0.75		
MgO	111	1.296	131.70	−1.24		

Materials and surface cuts higher up in the list in Table 4 that belong to both *l*(C–O) > 1.30 Å and OCO < 132° subgroups are the most promising catalysts, followed by materials that belong to one of the subgroups, with the performance decreasing further down the list. Taking into account the number of active surface cuts and Sabatier principle, we conclude that NaSbO_3_ is the most promising unexplored catalyst for temperatures up to 340 °C (for CO_2_ pressures around 1 atm). Other *A*^+1^*B*^+5^O_3_ type promising materials are KSbO_3_ (for temperatures up to 110 °C) and RbNbO_3_ (up to 360 °C) that belong to both subgroups, and LiSbO_3_ (230 °C), CsNbO_3_ (260 °C), CsVO_3_ (110 °C), NaVO_3_ (130 °C), belonging to one of the subgroups (listed in the order of decreasing performance). There are also several promising *A*^+3^*B*^+3^O_3_ oxides with surfaces belonging to one or both subgroups, listed in the order they appear first time in the table: ScAlO_3_ (up to 550 °C), GaAlO_3_ (230 °C), GaInO_3_ (340 °C), rhombohedral InAlO_3_ (120 °C)—these and other In-containing materials are of course very expensive, but we list them here for completeness, LaGaO_3_ (210 °C), ScGaO_3_ (240 °C), YAlO_3_ (330 °C).

From Table 4 it can be seen that not all promising materials belong to one of the found subgroups. This means that there are other optimal materials gene combinations that are not identified by SGD as statistically significant based on the current dataset. Such combinations may be unique for a given material, or they may be found when more data for different materials are considered. Among these materials the most promising are: InScO_3_ (up to 430 °C), MgSnO_3_ (430 °C), CaGeO_3_ (570 °C), orthorhombic InAlO_3_ (230 °C), CaSiO_3_ (420 °C), SrSiO_3_ (460 °C), SrGeO_3_ (480 °C), and BaSnO_3_ (up to 550 °C).

## Discussion

We have developed the subgroup-discovery strategy for finding improved oxide-based catalysts for the conversion of chemically inert molecules such as CO_2_ into useful chemicals or fuels. For this purpose we identified a new indicator of CO_2_ activation, namely the large C–O bond distance of the adsorbed molecule. This artificial-intelligence approach identifies the materials *genes* that correlate most strongly with the activation of the adsorbed molecule. Specifically, these are the following clean surface properties: Hirshfeld charges of O-atom at which CO_2_ adsorbs (*q*_O_) and of surface cations (*q*_min_, *q*_max_), surface geometric features [coordination descriptors *Q*_*i*_, *i* = 5, 6, distances between the surface O-atom and the nearest surface cations (*d*_*i*_, *i* = 1–3)], electrostatic potential and electric field above the adsorption site (Δφ, φ_2.6_), polarizability and *C*_6_ coefficients for surface atoms (*C*_6_^min^, *C*_6_^O^, α_max_), radii of HOMO and LUMO of the cation species (*r*_+1_^max^, *r*_+1_^min^, *r*_HOMO_^min^), ionization potential, electron affinity, and electronegativity of surface cation species (*IP*_max_, *EA*_max_, *EN*_min_), features of O 2*p* DOS (*kurt*, *M*, *PC*, *U*), conduction band minimum (*CBM*), energies of the lowest unoccupied projected eigenstates of surface cation species (*L*_max_, *L*_min_), and surface work function (*W*). The found subgroup selectors predict whether a given candidate material belongs to the class of promising catalysts. The peculiarity of the large C–O bond indicator is that it automatically satisfies Sabatier principle for low and middle-temperature CO_2_ conversion.

The present study shows also that the previously proposed indicator for CO_2_ activation, the decrease of the OCO-angle^27^, is not appropriate and even correlates with strong adsorption so that poisoning by carbonation is likely which may be useful for carbon capture and storage (CCS) but not for carbon capture and utilization (CCU). When Sabatier principle is purposely included in the SGD search for small OCO, found subgroups substantially overlap with large *l*(C–O) subgroups (70%), although still contain a few sites on inactive materials for CO_2_ conversion.

The subgroup analysis revealed an alternative mechanism of CO_2_ activation by adsorption, namely bonding of an O-atom in CO_2_ with a surface cation(s), combined with only moderate electron transfer from the surface to the molecule, which results not only in reduction of OCO-angles, but also in pronounced elongation and weakening of the C–O bond. Although the latter can be achieved also by a larger charge transfer, it results in stronger binding of CO_2_ molecule to the surface and poisoning of the catalyst, contrary to the new mechanism. The same new mechanism is revealed when Sabatier principle is included when searching for small OCO subgroups.

We also demonstrated that a standard regression technique (DTR), which gives prediction models in a physically interpretable form similar to subgroup discovery (selectors based on identified descriptor), fails to identify the optimal combinations of materials *genes* and the activation in general. This failure is traced back to the fact that DTR is a global approach, which minimizes error in the prediction of the value of a target property for the whole dataset. As a result, different combinations of *genes* leading to the optimal value of the same target property are intermixed, and the combination that leads to the most optimal value is not identified. On the contrary, subgroup discovery finds unique local subsets in the data independent of the rest of the data. This makes it more suitable for identifying different combinations of materials *genes* that result in activation.

The other four considered potential indicators (charge at the adsorbed CO_2_, adsorption induced dipole moment, the difference of charges on O-atoms and on C and O-atoms of adsorbed CO_2_) were found to reproduce the results of SGD obtained for OCO-angles or C–O bond distances with significant overlap with corresponding subgroups.

Based on our results, we propose several new promising oxide-based catalysts for CO_2_ conversion (Table 4). Although the present work has focused on oxides only, the overall strategy is general and can be applied to any other family of materials. This work also emphasizes the importance of documenting metadata and workflows for AI data analysis in materials science in order to ensure the reproducibility of AI models and data analysis results.

## Methods

### Ab initio calculations

The calculations are performed using density-functional theory (DFT) with the PBEsol exchange-correlation functional^55^ as implemented in FHI-aims code^56^ using ‘*tight*’ basis sets. The functional is chosen based on a comparison of calculated bulk lattice constants^55^ and CO_2_ adsorption energy to the available experimental results and high-level calculations (CCSD(T) and validated hybrid); see Supporting Information (SI) for more details on the computational setup. Nevertheless, it is expected that, because of the large set of systems inspected and the small variations introduced by the functional choice, the main trends will hold even when using another functional.

### Studied materials

The dataset includes 71 semiconductor oxide materials, with 141 surfaces. The materials are ternary (*AB*O_3_) and binary oxides with metal cations *A* and *B* from groups 1–5 (including La) and groups 12–15 of the periodic table. The full list of materials and surface cuts is given in Supplementary Notes, and the dataset is available in ref. ^26^. In this study we considered only stoichiometric surface reconstructions obtained by atomic relaxation of stoichiometric bulk-like initial surface geometries. While this seems to be a limitation, our results show that indicators of activation calculated with this assumption correlate with experimental activity for known good oxide catalysts. This does not imply that surfaces of these materials do not reconstruct, but that the properties of unreconstructed surfaces can be used as descriptors for catalysis at reconstructed and defected surfaces under realistic conditions. The inclusion of surface reconstructions in the training data will further improve the predictions and will be a subject of future work.

### The details of SGD

The SGD was done with the RealKD code (https://bitbucket.org/realKD/), modified to include quality functions described by Eqs. (1) and (2) in which the information gain was defined as:

3 $$u(p)=1-\left(\frac{-1}{{{{{{\rm{ln2}}}}}}}\right)(p\cdot \,{{{{\mathrm{ln}}}}}(p)+(1-p)\cdot \,{{{{\mathrm{ln}}}}}(1-p))$$

here *p* is the number of samples in a subgroup within the required adsorption energy range divided by the total number of samples in the subgroup. Since Shannon entropy is a symmetric parabola-like function around 0.5, we set here *F*(*Z*) = 0 for *p* ≤ 0.5. Also, *x·*ln(*x*) = 0 for *x* = 0. The search of subgroups is performed using a Monte-Carlo scheme adapted for these tasks^34^.

The cutoff values *x*, *y*, ... used for setting propositions (feature-1 < *x*, feature-2 ≥ *y*, etc.) are obtained by *k*-means clustering, as implemented within RealKD. That is, for a desired number *n* = *k* − 1 of cutoff values a set of *k* representative values of a given feature and *k* groups (clusters) of the data points are determined that minimize the deviation of all the feature values from the representative values. Thus, each value of the feature in the dataset is assigned to a particular cluster, and the cutoffs are determined as the arithmetic mean between the closest feature values in neighboring clusters. The number *k* is a parameter, and different *k*-values can in principle result in different cutoff values. It is worth noting that, due to the stochastic Monte-Carlo sampling, the exact definitions of the subgroups may vary for consecutive runs of the SGD algorithm. We have tested *k* = 12, 14, and 16 and rerun the algorithm several times for each *k*. While the results indeed depend on the run and on the *k* value, the subgroups maximizing the quality function have largely or entirely overlapping populations, and selectors with the same or similar propositions. Here we report selectors that appear most often and have high population and quality function values.

### Decision-tree regression

The DTR analysis was performed using Python scikit-learn libraries. DTR is a supervised learning method in which the training set is repeatedly split into patterns (so-called leaves) by means of propositions built from primary features. The fitting of a model is done with respect to the cost function, which encloses the deviation of fitted values of a target property from the actual values. In this study we considered two cost functions—mean squared error (MSE) and mean absolute error (MAE). The search for the most optimal partitioning (the so-called tree) is done with the greedy algorithm. To obtain the most optimal TR model, we used a standard approach for supervised machine learning—leave-one-out cross-validation with respect to the hyperparameters—minimal size of a leaf, maximal depth. The minimal size of a leaf is a bottom threshold of the population of a pattern, since too small size might result in overfitting. Maximal depth is a limit for the maximal number of splits in a tree.

## Supplementary information

Supplementary Information

Peer Review File

## Data Availability

The dataset is available in the NOMAD AI Toolkit^26^.

## Code availability

A Jupyter notebook is available in the NOMAD AI Toolkit^26^.
