# Supplementary material for: Artificial-intelligence-driven discovery of catalyst genes with application to CO2 activation on semiconductor oxides
Source: Nat Commun. 2022 Jan 20;13:419. doi: 10.1038/s41467-022-28042-z (PMC8776738; doi:10.1038/s41467-022-28042-z)
Supplement: Supplementary file 1 — Supplementary Information [file 41467_2022_28042_MOESM1_ESM.doc]

**Supplementary Information.**

**Artificial-intelligence-driven discovery of catalyst *genes* with application to CO_2_ activation on semiconductor oxides**

A. Mazheika^1^,*, Y. Wang^1^, R. Valero^2^, F. Viñes,^2^ F. Illas^2^, L. M. Ghiringhelli^1^, S. V. Levchenko^3,1^,*, M. Scheffler^1^

^1^Fritz-Haber-Institut der Max-Planck-Gesellschaft, 14195 Berlin-Dahlem, Germany

^2^Departament de Ciència de Materials i Química Física and

Institut de Química Teòrica i Computacional (IQTCUB),

Universitat de Barcelona, c/ Martí i Franquès 1, Barcelona 08028, Spain

^3^Skolkovo Institute of Science and Technology, Skolkovo Innovation Center, 3 Nobel Street, 143026 Moscow, Russia

*corresponding authors: [alex.mazheika@gmail.com](mailto:alex.mazheika@gmail.com), [mazheika@fhi-berlin.mpg.de](mailto:mazheika@fhi-berlin.mpg.de); [levchenko@fhi-berlin.mpg.de](mailto:levchenko@fhi-berlin.mpg.de)

**Supplementary methods**

***Ab initio* methods.** All *ab initio* calculations were performed with the all-electron full-potential electronic structure FHI-aims code package^1^ using density-functional theory (DFT) and numerical atom-centered basis functions. The standard 'tight' settings (grids and basis functions) were employed^1^, which deliver the adsorption energies with basis-set superposition errors below 0.07 eV per adsorbed molecule. The exchange-correlation (XC) functional approximation was chosen based on a comparison to available experimental and high-level theoretical data on CO_2_ adsorption energy (see below). PBE^2^ and PBEsol^3^ functionals with and without Tkatchenko-Scheffler (TS) pairwise dispersion-correction method^4^ were tested. LDA^5^ and RPBE^6^ have been previously shown to give large errors for adsorption of CO_2_^7,8^. All systems were treated as spin non-polarized. The bulk lattice vectors were calculated with the same exchange-correlation functional as the surface and the adsorbed molecule properties. The *k*-points for the bulk calculations were converged with respect to lattice vectors. The slabs were symmetric, and all atoms therein were allowed to relax. We did not constrain any side of a slab in order to have the same surface geometry on both sides, which is important for calculation of surface primary features. The slab thickness was also tested, and it was set to about 11 Å or larger in most cases, based on the convergence of the surface energy (within 5 meV/Å^2^) and the work function (within 10 meV) with respect to the thickness. For the surface supercells the *k*-grids were scaled from corresponding bulk grids. The lattice constants were obtained from the relaxed bulk unit cells. The initial geometries of adsorbed CO_2_ before full atomic relaxation were obtained by placing the CO_2_ molecule at different possible adsorption sites (metal and O sites, top, bridge, and hollow sites) and in different orientations (C down, O down) on one side of the slab. The size of the surface supercells was set based on test calculations, so that the interaction between the periodic images of the adsorbed CO_2_ was below 0.1 eV. The resulting distance between the images of the C atom was about 8 Å. The adsorption of CO_2_ has been considered only on one side of the slab, and a dipole correction^9^ was included to prevent spurious electrostatic interactions. The lattice vector along the direction parallel to the vacuum gap was 200 Å. All atoms in the systems have been allowed to relax until the maximum remaining force fell below 10^-2^ eV/Å.

There are few experimental data available for CO_2_ adsorption at clean monocrystalline surfaces without impurities: at CaO (001)^10^ and at ZnO (10-10)^11,12^. We compared the calculated adsorption energies (*E*_ads_) to the microcalorimetry and temperature programmed desorption (TPD) data. The adsorption energies were calculated as the difference between total energies of the slab with the adsorbed molecule, clean surface slab, and a free gas-phase CO_2_ molecule. The calculations of the surfaces were performed with symmetric 5-atomic layer slabs for CaO (001) and 4 double-layer slab for ZnO (10-10). 8×8×8 and 10×10×6 *k*-point grids were used for cubic CaO and hexagonal ZnO bulk unit cells, respectively. Surface unit cells were (2×2) for CaO (001), for ZnO (10-10) we considered two cells – (1×1) and (1×2).

The results for CaO (001) and ZnO (10-10) are shown in Supplementary Table 1. In the case of CaO the PBE adsorption energy is the closest to the experimentally observed value both from TPD and microcalorimetry, whereas PBEsol and PBEsol+TS values are closer to the one obtained with CCSD(T) using an embedded cluster model^10^. The inconsistency of the high-level theoretical and the experimental results was explained^10^ by the formation of agglomerates of adsorbed CO_2_ molecules even in ultrahigh vacuum. Relative to CCSD(T), PBEsol+TS performs better.

In the case of ZnO (10-10) the experimental data have been obtained for two adsorption coverages: 100% [(1×1) structure] and 50% [(1×2) structure]. In contrast to CaO, TPD and microcalorimetry values differ by about 0.2 eV (Supplementary Table 1). Taking into account that the calculated thermo-desorption energies depend on the chosen kinetic model as well as on the pre-exponential factor, we consider the microcalorimetry results as more accurate. The PBEsol adsorption energies match both measured values with the best accuracy (~0.1 eV). PBEsol+TS slightly overestimates the adsorption energies. This is not unexpected, since PBEsol functional behaves similarly to LDA for interatomic interactions at the middle-range distances, so that inclusion of additional vdW-correction leads to overestimation of binding energies. In addition, the TS scheme based on non-iterative Hirshfeld partitioning of the electron density was found to fail in predicting adsorption energies for some ionic systems, due to inaccurate description of polarizabilities^13^.

**Supplementary Table 1.** The experimental and theoretical energies of adsorption (in eV) of CO_2_ at CaO (001) and ZnO (10-10) surfaces.

| method | CaO (001) | ZnO (10-10) | | MgO (001) |
| --- | --- | --- | --- | --- |
|  |  | (1×1) structure | (1×2) structure |  |
| PBE | -1.32 | -0.45 | -0.67 | -0.34 |
| PBE+TS | -1.47 | -0.79 | -0.96 | -0.53 |
| PBEsol | -1.60 | -0.84 | -1.04 | -0.63 |
| PBEsol+TS | -1.75 | -1.00 | -1.19 | -0.79 |
| TPD | -1.24 – -1.45 [10] | -0.55 [11] | -0.90 [11,12] | -0.41 [14] |
| microcalorimetry | ~ -1.30 [10] | -0.72 [12] | -1.12 [12] | - |
| high-level calculations | -1.91 ± 0.10^a^ [10] | - | - | -0.64^b^ [15] |

^a^CCSD(T); ^b^HSE(0.3)+vdW

We also compare the GGA CO_2_ adsorption energies for MgO (001) surface with hybrid HSE(0.3)+vdW functional results^15^, where HSE(0.3)+vdW is the HSE functional with 30% fraction of exact exchange plus the many-body dispersion correction^16^. This functional was shown to yield CO_2_ adsorption energies very close to CCSD(T) for embedded clusters^15^, and the adsorption energy was found to be -0.64 eV. The closest value was obtained with the PBEsol functional (-0.63 eV). Thus, PBEsol compares favorably to both experiment and higher-level calculations. In addition to the above-mentioned systems, two more systems were tested: CO_2_ adsorption on BaO-terminated BaTiO_3_ (001) and on CaZrO_3_ (101) surfaces. In general, we find that *relative* *differences* in adsorption energy between different XC approximations are weakly dependent on the material and surface termination (Supplementary Figure 1, left).

In addition to adsorption energies, another important parameter of CO_2_ adsorption is the OCO angle, which is 180º in the neutral gas-phase molecule and close to 120º (as in a gas-phase CO_3_^2-^ ion) in adsorbed systems. As there are no precise experimental data like in the case of adsorption energies, here we rely on a weak sensitivity of the OCO angle to XC functional approximations. PBE, PBE+TS, and PBEsol provide very close OCO-angles for all tested systems (Supplementary Figure 1, right). The largest difference was observed in MgO (001) case where PBE+TS value is larger than PBE and PBEsol by 1.0°. In all other cases such deviation was 0.4 degree on average.


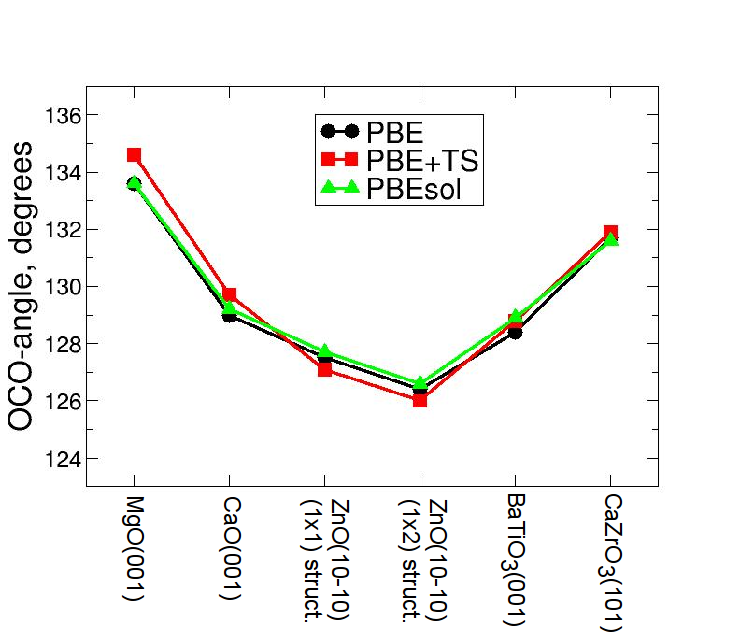


**Supplementary Figure 1.** The adsorption energies (left) and OCO-angles (right) of adsorbed CO_2_ for different surfaces and XC functionals.

Summarizing the test results and taking into account that PBEsol provides a very good agreement between calculated and experimental bulk lattice constants for ionic solids^3^, we conclude that PBEsol is the best choice for our study. This result may be explained by the accuracy in prediction of lattice parameters in PBEsol^17^ that results in a correct distribution of electronic density on surfaces.

**Decision tree regression models obtained for *l*(C-O).** We have done a comparison of found SGD subgroups with DTR performance for *l*(C-O). Two cost functions were used in DTR – mean absolute error (MAE) and mean squared error (MSE), and the patterns with largest average *l*(C-O) values in each obtained tree were analyzed. We did not take into account the Sabatier principle explicitly in DTR, since there exists no standard decision tree algorithm wrapping regression and classification simultaneously. Thus, we do not consider DTR for OCO-angle.

The leave-one-out cross validation was used for the search of the optimal set of hyperparameters – minimal size of a leaf and maximum depth of the tree. The search was done on a grid at first for the minimal size of a leaf, then for selected local and global minima for maximum depth of the tree with fixed minimal sizes. The most optimal sets of hyperparameters {min. size, max. depth} are {27, 4} for *l*(C-O) DTR model with MSE cost function and {60, 2} for *l*(C-O) DTR model with MAE (Supplementary Figure 2). With these sets of hyperparameters the regression trees shown in Supplementary Figure 3 were obtained.

**Supplementary Figure 2**. The results of leave-one-out cross-validation for tree regression models with respect to the minimal size of a leaf and next the maximum depth of the tree (insets): a) *l*(C-O) as the target with MSE as the cost function; b) *l*(C-O) as the target with MAE as the cost function.

**Supplementary Figure 3**. The regression tree models obtained for a) OCO angle as the target with MSE as the cost function; b) OCO angle as the target with MAE as the cost function.

The DTR patterns with the largest mean value are dependent on which cost function is used. With MSE as cost function, the pattern with large *l*(C-O) values is defined as (*EA*_max_ ≤ -0.24 eV) AND (α_max_ > 94.80) AND (Δφ > 0.43 eV), with 39 samples and 1.323 Å average; with MAE, the corresponding pattern is defined as (*EA*_max_ ≤ -0.037 eV) AND (*q*_min_ ≤ 0.47 *e*), with 61 samples and 1.308 Å average. Both patterns significantly exceed the size of the *l*(C-O) > 1.30 Å subgroups and contain many samples in common. Among samples not present in both patterns, the pattern obtained with MSE has all adsorption sites on La_2_O_3_, and the pattern from MAE cost function has sites on Na_2_O. Both lanthanum and sodium oxide are materials prone to extremely strong carbonation (Supplementary Table 2). Moreover, the pattern obtained with MAE cost function contains two sites above which CO_2_ prefers to physisorb, with *l*(C-O) = 1.17 Å. This clearly demonstrates the tendency of DTR to overemphasize the importance of data points based solely on the value of target property. DTR minimizes the overall cost function, so that the local regularities are not explicitly considered and are smeared out for the sake of optimizing the global fit, whereas the SGD with quality function (2) is exactly focused on revealing such local subsets. As a result, materials with sites where C-O bond is strongly elongated due to a large charge transfer and the sites above which CO_2_ is not activated are selected by DTR together with materials providing moderate charge transfer, but at the same time additional bonding of O atom in adsorbed CO_2_ with a surface cation, which also leads to C-O bond elongation. Thus, DTR in this case fails to distinguish these two very different activation modes and, in some cases, cannot even distinguish activation from non-activation.

**Supplementary notes.**

**Studied materials and surface terminations.** In the current study we have focused on semiconductor (or insulating) oxide materials. Furthermore, we do not consider defects (*e.g.*, oxygen vacancies) and charge-carrier doping, which can significantly modify surface chemical properties. Despite these constraints, the selected materials class includes a large number of compounds (binary, ternary, and more complex oxides). Metallic oxides and defects on surfaces will be the object of the next study. In general, three groups of oxides have been considered: *A*^2+^*B*^4+^O_3_, *A*^1+^*B*^5+^O_3_, *A*^3+^*B*^3+^O_3_ and all the binary oxides *A*O, *B*O_2_, *A*_2_O_3_, *A*_2_O, *B*_2_O_3_. For each oxide material we have considered a set of low-index surfaces with maximal Miller index up to 2. We mainly considered non-polar surfaces. For several included polar surfaces, reconstructions that compensate surface charge assuming formal charges of the ions were considered. All surfaces were insulating (with a non-vanishing gap between the highest occupied and lowest unoccupied states). In the cases when oxides have polymorphs (TiO_2_, MgGeO_3_ etc.) they were also included. The full list of materials and surface terminations is shown in Supplementary Table 2. In general, 71 materials have been calculated with 141 surfaces including different terminations. Considering all non-equivalent adsorption sites on these surfaces, the total number of calculated unique CO_2_ adsorption geometries is 255. All data, including initial and final geometries, and the computed properties, are available in the NOMAD database^16^.

**Supplementary Table 2.** Oxide materials, surface terminations, and the number of unique adsorption sites per termination.

| material | surfaces | number of unique sites per surface |
| --- | --- | --- |
| MgSiO_3_ | (001) MgO-term. | 1 |
| MgTiO_3_ | (001)  (012) | 1  2 |
| MgGeO_3_ hexagon. | (001)  (012) | 1  2 |
| MgGeO_3_ tetragon. | (001) MgO-term.  (001) GeO_2_-term. | 1  1 |
| MgSnO_3_ | (100) | 2 |
| CaSiO_3_ | (001) CaO-term.  (001) SiO_2_-term.  (110) CaO-term.  (110) SiO_2_-term. | 1  1  1  1 |
| CaTiO_3_ | (010) CaO-term.  (101) CaO-term.  (100) TiO_2_-term. | 1  1  1 |
| CaGeO_3_ | (001) CaO-term.  (001) GeO_2_-term.  (110) CaO-term.  (110) GeO_2_-term. | 1  2  1  2 |
| CaZrO_3_ | (010) CaO-term.  (101) CaO-term.  (101) ZrO_2_-term. | 1  2  1 |
| CaSnO_3_ | (001) SnO_2_-term.  (110) CaO-term.  (110) SnO_2_-term. | 1  2  1 |
| SrSiO_3_ | (001) SrO-term. | 1 |
| SrTiO_3_ | (001) SrO-term.  (001) TiO_2_-term. | 1  1 |
| SrGeO_3_ | (100) SrO-term.  (100) TiO_2_-term. | 1  1 |
| SrZrO_3_ | (001) ZrO_2_-term.  (110) SrO-term. | 1  2 |
| SrSnO_3_ | (001) SrO-term.  (001) SnO_2_-term.  (110) SrO-term.  (110) SnO_2_-term. | 1  1  1  1 |
| BaSiO_3_ | (100)  (101) | 2  1 |
| BaTiO_3_ | (001) BaO-term.  (001) TiO_2_-term. | 1  1 |
| BaGeO_3_ | (001) BaO-term. | 1 |
| BaZrO_3_ | (001) ZrO_2_-term.  (110) BaO-term. | 1  1 |
| BaSnO_3_ | (001) BaO-term.  (001) SnO_2_-term. | 1  1 |
| MgO | (001)  (110)  (111) octopolar O-term. | 1  1  1 |
| CaO | (001)  (110)  (111) octopolar O-term. | 1  1  1 |
| SrO | (001)  (110)  (111) octopolar O-term. | 1  1  1 |
| BaO | (001)  (110)  (111) octopolar O-term. | 1  1  1 |
| SiO_2_ | (001) | 2 |
| TiO_2_ anatase | (101)  (001) | 2  1 |
| TiO_2_ rutile | (100)  (110) | 1  2 |
| GeO_2_ | (100)  (110) | 1  2 |
| ZrO_2_ | (001)  (011)  (111) | 2  4  3 |
| SnO_2_ | (100)  (110) | 1  2 |
| ZnO | (10-10) | 1 |
| LiNbO_3_ | (100) | 1 |
| NaNbO_3_ tetragon. | (010)  (110) | 2  1 |
| NaNbO_3_ P bcm | (100) | 1 |
| KNbO_3_ tetragon. | (010)  (110) | 1  2 |
| RbNbO_3_ P1 | (111) | 2 |
| CsNbO_3_ | (010)  (100) | 2  1 |
| LiVO_3_ orthogon. | (110) | 2 |
| LiVO_3_ P bcm | (100) | 1 |
| NaVO_3_ | (010)  (110) | 1  1 |
| KVO_3_ orthogon. | (010) | 1 |
| RbVO_3_ tetragon. | (010)  (110) | 1  1 |
| RbVO_3_ P bcm | (100) | 1 |
| CsVO_3_ tetragon. | (010)  (110) | 1  1 |
| LiSbO_3_ tetragon. | (010) | 1 |
| LiSbO_3_ P bcm | (100) | 1 |
| NaSbO_3_ tetragon. | (010) | 1 |
| NaSbO_3_ P bcm | (100) | 2 |
| KSbO_3_ tetragon. | (110) | 2 |
| Na_2_O | (011)  (111) | 1  1 |
| GaAlO_3_ | (100) | 2 |
| InAlO_3_ hexagon. | (110) | 2 |
| InAlO_3_ orthorh. | (010)  (110)  (121) | 3  4  3 |
| GaInO_3_ | (100)  (110)  (120) | 2  5  6 |
| ScAlO_3_ | (010)  (100)  (110)  (121) | 1  2  2  6 |
| ScGaO_3_ | (010)  (110) | 3  5 |
| ScInO_3_ | (100)  (110) In_2_O_3_-term.  (110) ScInO_3_-term.  (121) | 5  5  5  6 |
| YScO_3_ | (100) | 1 |
| LaScO_3_ | (100) | 1 |
| YInO_3_ | (100)  (110) | 2  2 |
| YAlO_3_ | (011)  (100) | 2  1 |
| LaYO_3_ | (001) | 2 |
| YGaO_3_ | (100)  (110) | 2  2 |
| LaAlO_3_ | (110) | 2 |
| LaGaO_3_ | (100)  (110) | 1  1 |
| LaInO_3_ | (100) | 1 |
| Al_2_O_3_ | (001)  (012) | 1  1 |
| Ga_2_O_3_ | (110)  (212) | 3  7 |
| Sc_2_O_3_ | (001)  (110)  (111) | 3  5  5 |
| In_2_O_3_ | (001)  (110)  (111) | 1  5  4 |
| La_2_O_3_ | (100)  (110)  (120)  (201) | 2  2  3  2 |

**Supplementary Figure 4.** The dependence of LUMO radii (*r*_+1_) on electron affinities. Red dashed lines show isovalues *r*_+1_ = 1.94 Å and 2.80 Å.

**Supplementary Table 3.** The full list of used primary features calculated with PBEsol.

| symbol | meaning |
| --- | --- |
| *IP*_min/max_, *IP*_O_ | ionization potential, minimal and maximal in the pair of atoms *A* and *B*, and for O; calculated as *E*_atom_ - *E*_cation_ |
| *EA_min/max_*, *EA*_O_ | electron affinity, minimal and maximal in the pair of atoms *A* and *B*, and for O; calculated as *E*_anion_ - *E*_atom_ |
| *EN*_min/max_, *EN*_O_ | Mulliken electronegativity, minimal and maximal in the pair of atoms *A* and *B*, and for O |
| *r*_HOMO_, *r*_+1_*, r*_-1_ | maximum value of radial wave functions of the non-spin polarized spherically symmetric atom for HOMO, LUMO and HOMO-1 |
| Δ | band gap of the whole surface slab |
| *E*_form_ | surface formation energy |
| *VBM* | valence-band maximum with respect to vacuum level |
| *W* | work function (*W* = -*VBM*) |
| *q*_O_ | Hirshfeld charge of O-atom |
| *q*_min_, *q*_max_ | minimal and maximal Hirshfeld charges of cations in the pair *A* and *B*, calculated as an average for all surface cations of a given type |
| φ_1.4_, φ_2.6_, φ_1.4_ - φ_2.6_ | electrostatic potentials above O-atom at 1.4 and 2.6 Å and their difference. 1.4 Å corresponds to the average length of the bond between C and surface O, 2.6 Å is the minimal distance from surface O to C-atom of physisorbed carbon-dioxide molecule as observed from our calculations |
| *α*_O_, *C*_6_^O^ | polarizability and *C*_6_-coefficient for O-atom obtained from many-body dispersion scheme [16] |
| α_min_, α_max_, *C*_6_^min^, *C*_6_^max^ | polarizability and *C*_6_-coefficient for cations, minimal and maximal in the pair *A* and *B*, calculated as an average for all surface cations of a given type |
| *Q*_5_, *Q*_6_ | local-order parameter with *l* = 5 or 6 |
| *d*_1_, *d*_2_, *d*_3_ | distances from surface O-atom to the first-, second-, and third-nearest cations |
| *BV* | bond-valence value of O-atom |
| *PC* | weighted O 2*p*-band center |
| *c*_min_, *c*_max_ | first moment for PDOS of cation within valence-band, minimal and maximal in the pair *A* and *B*, calculated as an average for all surface cations of a given type |
| *wid* | square-root of the second moment of O 2*p*-band |
| *wid*_min_, *wid*_max_ | square-root of the second moment for PDOS of cations within valence-band, minimal and maximal in the pair *A* and *B*, calculated as an average for all surface cations of a given type |
| *skew* | skewness of O 2*p*-band PDOS |
| *kurt* | kurtosis of O 2*p*-band PDOS |
| *CBm* | conduction band minimum |
| *L*_min_, *L*_max_ | energy of lowest unoccupied state of cation, minimal and maximal in the pair *A* and *B*, calculated as an average for all surface cations of a given type |
| *M* | energy at which the O 2*p*-band PDOS is maximal |
| *U* | eigenstate with least negative value in O 2*p*-band |

**Supplementary Table 4.** Top subgroups obtained by minimization of OCO-angle with/out energy constraint and corresponding distributions of samples according to adsorption energies, OCO-angles, and C-O bond distances.

| cutoff | size | selector |
| --- | --- | --- |
| without adsorption energy constraint | | |
| 123 | 9 | q_O_>=-0.39, Q_5_<=0.81, Δ>=1.675, PC>-6.61, q_min_<=0.58 |
| 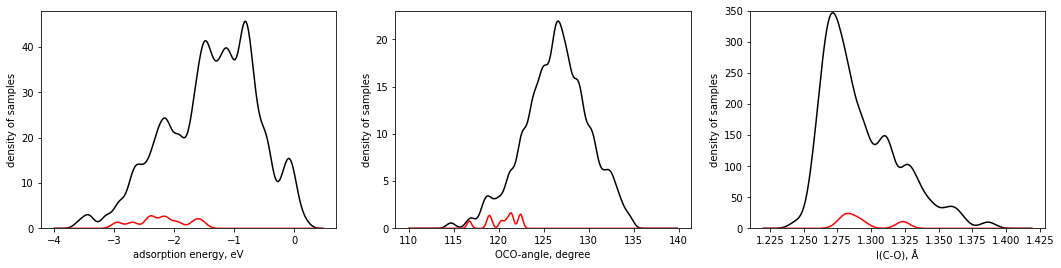 | | |
| 124 | 14 | L_min_>-1.76, Q_5_>=0.69, α_max_>100.4, c_max_<=-6.00, α_O_<=1.63 |
| 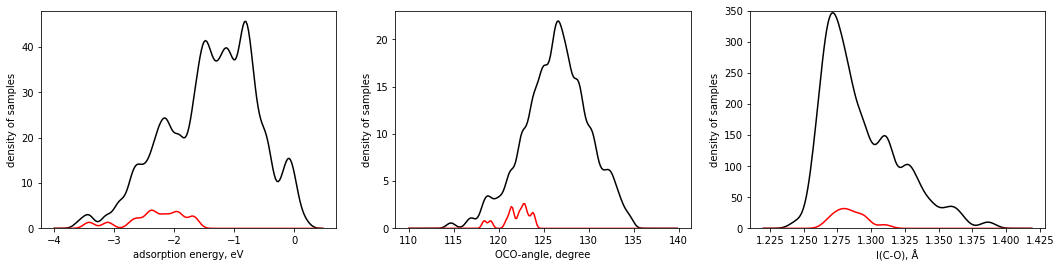 | | |
| 126 | 19 | *L*_max_>-2.70, *IP*_max_≥-6.05, α_max_≤184.5, Δφ>1.33, *q*_max_≤0.59, *wid*≤1.59, *wid*≥0.58 |
| 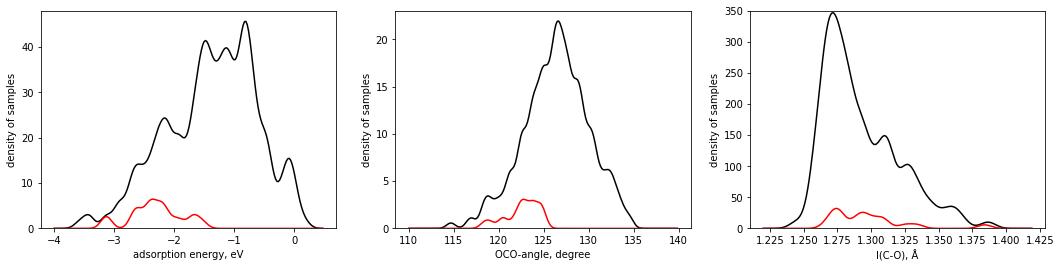 | | |
| 128 | 44 | EA_max_>=-0.425, Q_6_>=0.51, α_max_>=50.4, Δφ>=1.00, q_min_<=0.49 |
| 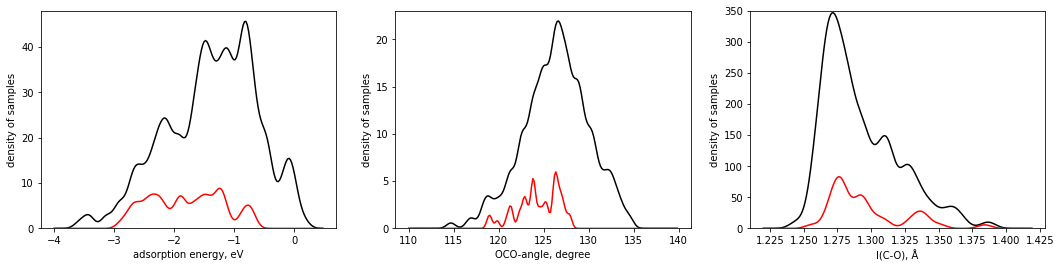 | | |
| 130 | 77 | L_max_>=-5.23, EA_max_<=0.16, d_1_>=1.82, d_2_>2.10 |
| 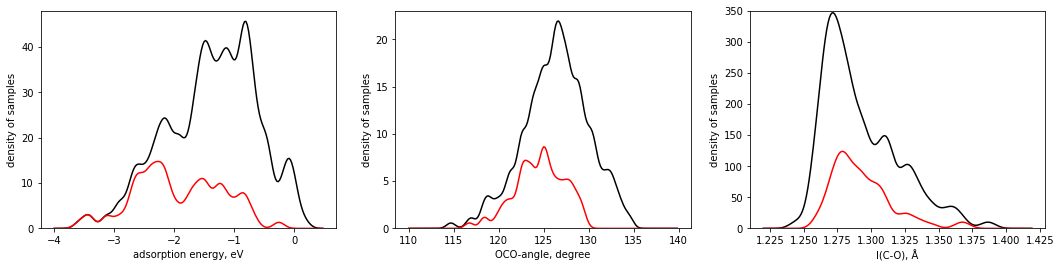 | | |
| 132 | 139 | IP_max_>=-6.99, q_O_<=-0.32, C_6_^O^>=10.36 |
| 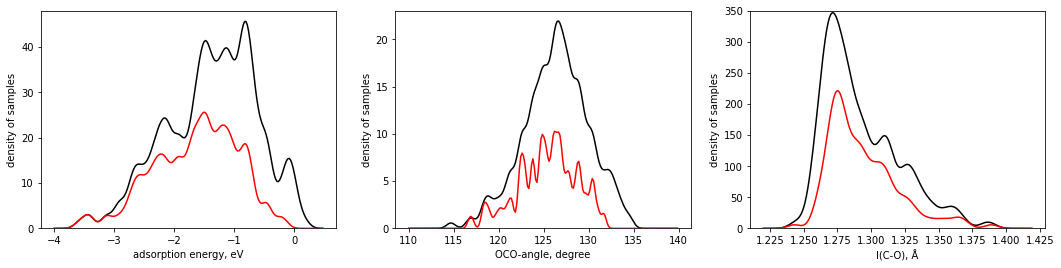 | | |
|  | | |
| with adsorption energy constraint | | |
| 123 | 8 | Q_6_<0.66, c_max_>=-9.80, d_2_>=2.00, M<=-4.12, wid_min_>1.52 |
| 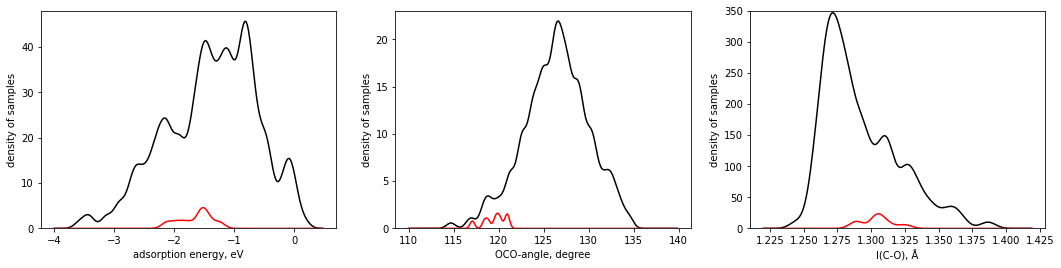 | | |
| 124 | 10 | *q*_O_<=-0.32, Q_6_<0.66, Q_6_>=0.57, Δφ>=0.60, r_-1_^max^<=1.65, wid>=1.24 |
| 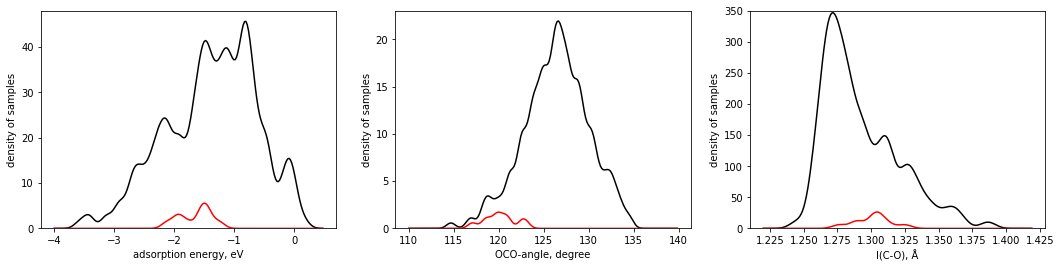 | | |
| 126 | 15 | *L*_min_≥-5.1085, φ_2.6_≤0.3033, Δφ≤1.0622, *d*_1_≥1.82, *d*_2_≥2.005, r_+1_^max^>2.83 |
| 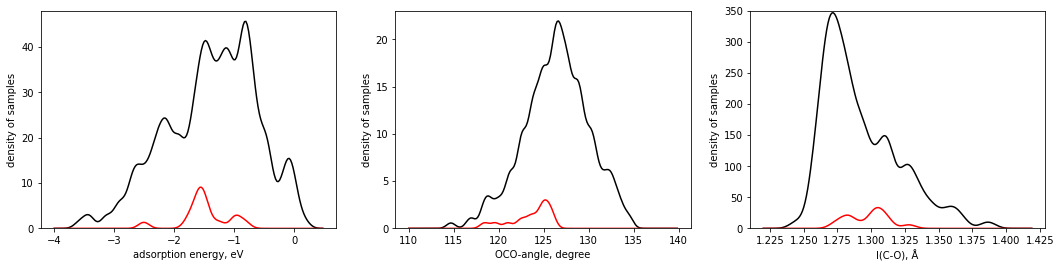 | | |
| 128 | 30 | C_6_^min^>=369.5, *L*_max_>=-4.73, Q_5_<=0.83, Δφ>=0.60, r_+1,max_>=2.80, C_6_^O^<=12.10 |
| 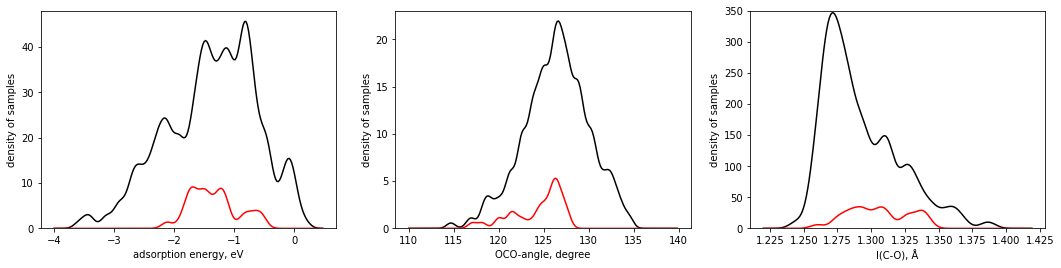 | | |
| 130 | 40 | φ_2.6_>=-0.15, Δφ>=0.73, d_1_<=2.01, d_2_>=1.96, d_3_>=2.025, q_min_<=0.49, r_+1_^min^>=1.94 |
| 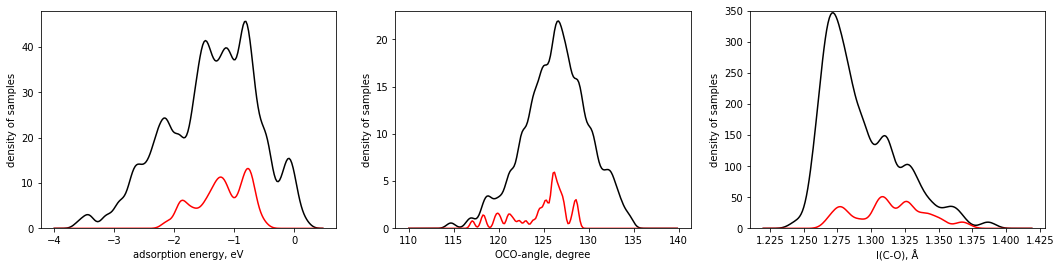 | | |
| 132 | 58 | *q*_O_≤-0.3386, *M*≤-6.292, *kurt*≥2.1035, *IP*_max_≥-6.2085, *r*_HOMO_^min^≤1.407 |
| 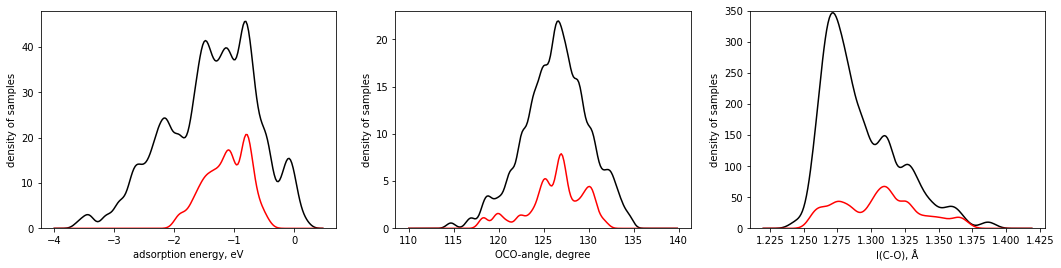 | | |

**Supplementary Table 5.** Top subgroups obtained by maximization of *l*(C-O)-bond with/out energy constraint and corresponding distributions of samples according to adsorption energies, OCO-angles, and C-O bond distances.

| cutoff | size | selector |
| --- | --- | --- |
| without adsorption energy constraint | | |
| 1.26 | 121 | C_6_^min^>=343.5, φ_2.6_<=0.66, Q_5_<=0.83, M>=-8.05 |
| 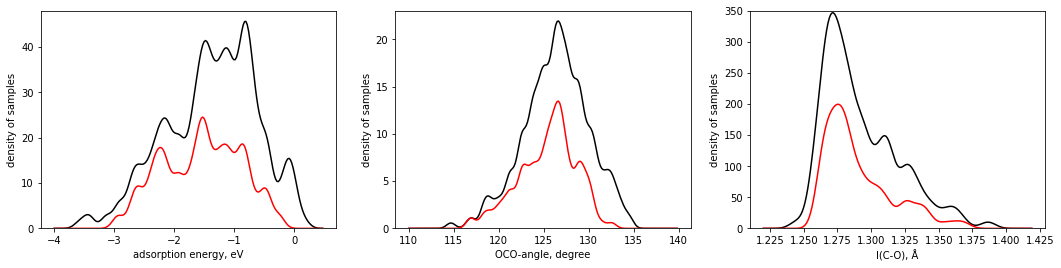 | | |
| 1.28 | 38 | EA_max_<=0.005, d_2_>2.22, M<=-4.12 |
| 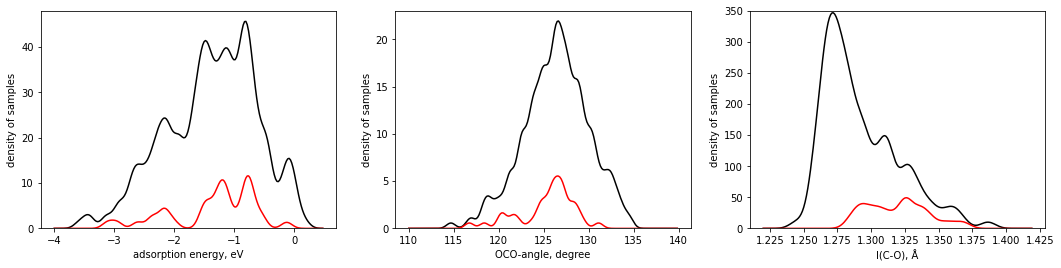 | | |
| 1.30 | 27 | kurt>=2.10, d_2_>2.14, U<=-5.34, q_min_<0.48 |
| 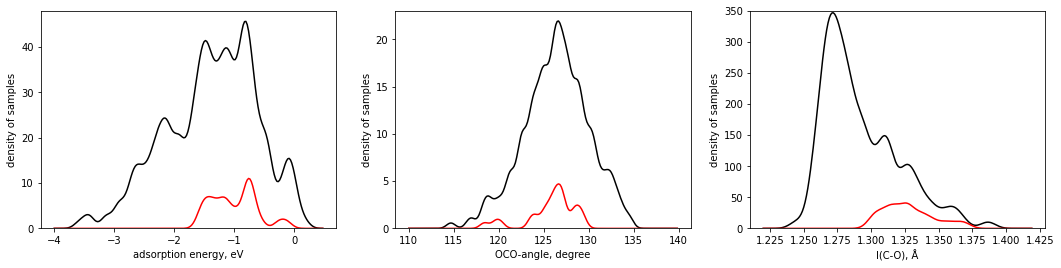 | | |
|  | | |
| with adsorption energy constraint | | |
| 1.26 | 56 | CBM>=-5.17, Δφ<=1.13, *PC*>=-8.62, d_3_<=2.48, M<=-6.06 |
| 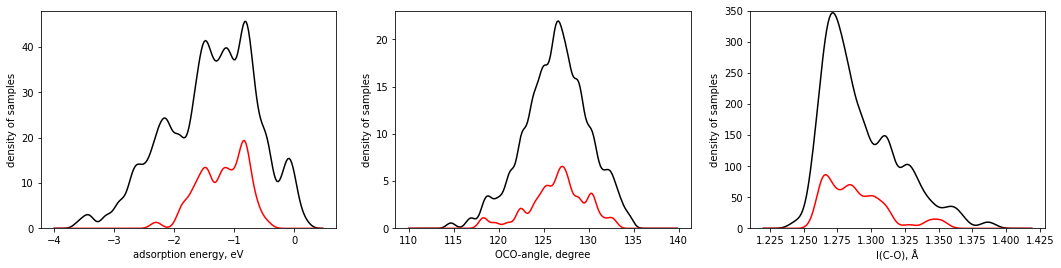 | | |
| 1.28 | 30 | W>=5.1, d_2_>2.14, q_min_<0.48 |
| 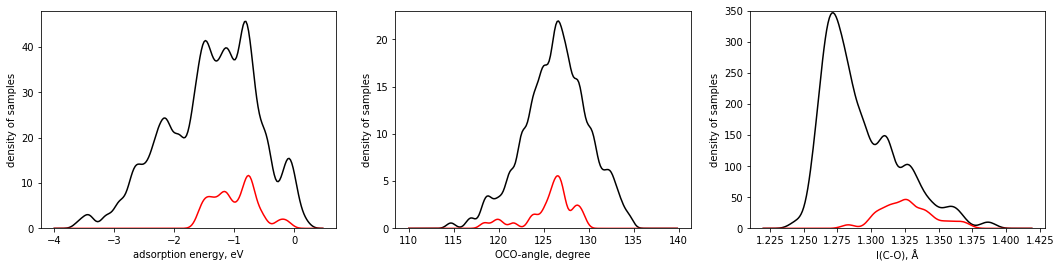 | | |
| 1.30 | 27 | EA_max_<=0.005, EN_min_<=-3.19, kurt>=2.51, d_2_>2.14, q_min_<0.48 |
| 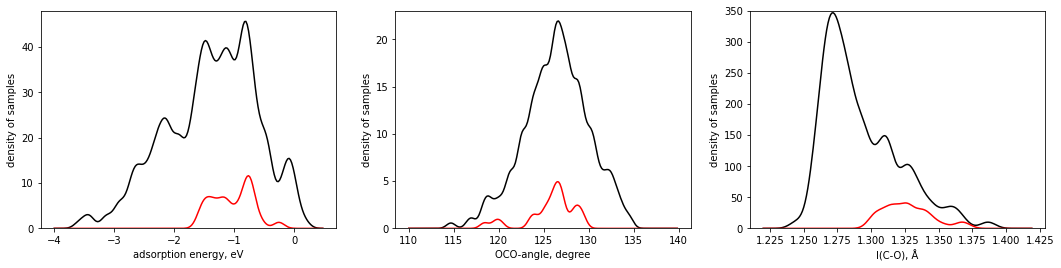 | | |


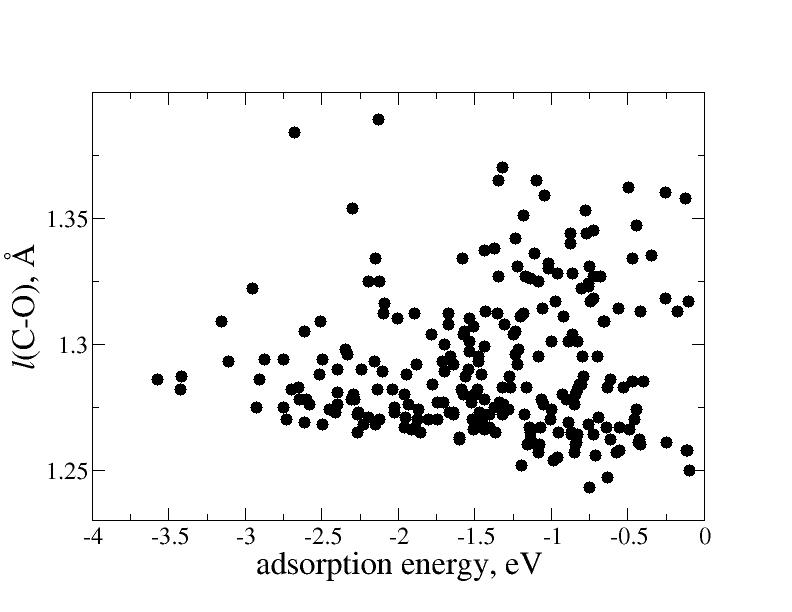
 **
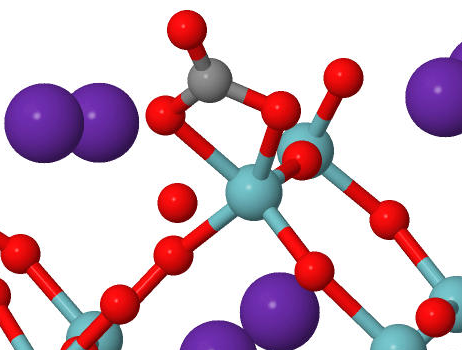
**

**Supplementary Figure 5**. (left) The dependence of CO_2_ adsorption energy on C-O bond length *l*(C-O). (right) Typical CO_2_ adsorption structure from the subgroup with larger *l*(C-O). Color scheme: gray C, red O, cyan Nb, violet Rb.

**Supplementary discussion.**

**1. Dipole moment of the slab induced by adsorbed CO_2_ molecule.** The dipole moment of the slab with adsorbed CO_2_ molecule indicates both the bending of the molecule and the amount of charge transferred to the molecule upon adsorption (the dipole moment of the slab before adsorption is zero, since we use symmetrically terminated slabs), and thus it indicates the molecule activation. Since in our models the CO_2_ adsorption was considered on one side of a surface slab, the dipole moment can be calculated as the difference of electrostatic potentials in vacuum at the two sides of the slab normalized per the surface area. The distribution of the calculated dipole moments in our data shows that certain number of samples has a positive dipole moment (Supplementary Figure 6, left), which is the result of surface relaxation upon CO_2_ adsorption. We have performed SGD with the minimization of the dipole moment (eq. 1 of the main text), which corresponds to a larger amount of electron density transferred to the CO_2_ molecule. Three thresholds have been chosen – -0.002, -0.005, and -0.008. For the cases with both Sabatier principle constrain and without it obtained subgroups are shown in Supplementary Table 6.

The distribution of adsorption energies for obtained subgroups is shown in Supplementary Figure 6 left. In all cases where no Sabatier principle constraint was introduced in the quality function there are samples for which strong carbonation is observed with adsorption energies around -3 eV.

Among subgroups obtained with Sabatier principle constraint the one with -0.002 threshold is mostly populated (Supplementary Table 6). It contains adsorption sites on several mentioned in the main text good catalysts – LaAlO_3_, Ga_2_O_3_, but also on a less promising YInO_3_. Regarding other materials from this subgroup there is no reliable information.

**Supplementary Table 6.** The subgroups obtained for SGD minimization of a dipole moment.

| threshold | size | subgroup |
| --- | --- | --- |
| Dipole minimization without Sabatier principle constraint | | |
| -0.002 | 57 | *d*_1_≤2.2025, *d*_3_≤2.9045, *U≥*-6.108, *r*_+1_^max^≤2.8315 |
| -0.005 | 23 | *L*_min_>-2.19, *EA*_max_≥-0.464, *Q*_5_≤0.8113, *Q*_6_≤0.7756, *r*_-1_^max^≤1.652 |
| -0.008 | 5 | *L*_min_>-2.538, *EA*_max_≤0.157, *d*_1_<1.8635, *d*_2_<2.037, *r*_+1_^min^≤2.093 |
|  | | |
| Dipole minimization with Sabatier principle constraint | | |
| -0.002 | 46 | *CBM*≥-5.1675, *q*_O_≥-0.3906, *Q*_5_≥0.51525, *VBM*≤-5.7975, *M*≥-7.285, *q*_max_≤0.64775, *r*_HOMO_^min^≥0.581 |
| -0.005 | 12 | *L*_min_>-2.19, *EN*_min_≤-3.275, *r*_+1_^min^≤2.807, *wid*≥1.242 |
| -0.008 | 8 | φ_2.6_≤0.66395, *IP*_min_≤-5.831, *c*_min_>-9.361, *kurt*≤8.576, *q*_min_<0.43575 |

**
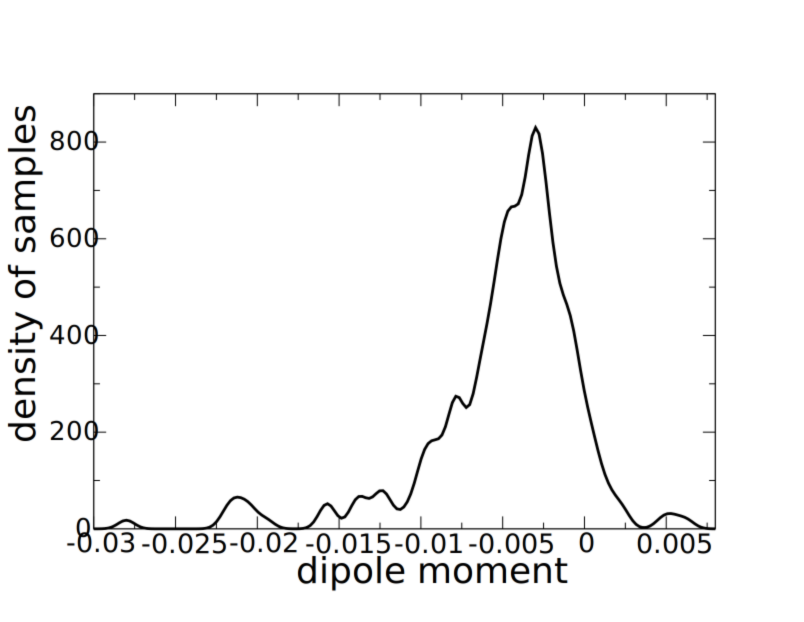
**

**Supplementary Figure 6**. (left) The distribution of samples according to the calculated dipole moment in the whole data set. (right) The distribution of adsorption energies in obtained subgroups.

**2. Hirshfeld charge of an adsorbed CO_2_.** The physical reasoning behind this indicator is the same as in the case of the dipole moment. Although partitioning of electron density among atoms in a solid is not uniquely defined, different partitioning schemes and in particular Hirshfeld partitioning^18^ qualitatively capture changes in electron distribution. SGD was performed with quality function shown in eq. 1 of the main text. Three thresholds were considered – -0.1, -0.2 and -0.3 *e*. Obtained SGD subgroups are shown in Supplementary Table 6.

The distribution of adsorption energies for corresponding subgroups is presented in Supplementary Figure 7. For the unconstrained case there is again a domain of samples with large absolute values of adsorption energies. All subgroups obtained with and without Sabatier principle constraint overlap significantly with reduced OCO subgroups. For example the overlap between unconstrained OCO < 132° and *q*(CO_2_) < 0.1 *e* subgroups is 91% and 74% of the population respectively, and for corresponding Sabatier principle constrained subgroups – 69 % and 49%.

**Supplementary Table 6.** The subgroups obtained for SGD minimization of adsorbed CO_2_ Hirshfeld charge – *q*(CO_2_).

| threshold | size | subgroup |
| --- | --- | --- |
| *q*(CO_2_) minimization without Sabatier principle constraint | | |
| -0.1 | 171 | *q*_O_≤-0.3386, *Q*_6_≤0.9458, Δ≤4.07 |
| -0.2 | 72 | φ_1.4_≥1.051, *Q*_5_≤0.82885, *E*_form_<0.077 |
| -0.3 | 22 | *IP*_min_<-6.4695, *IP*_max_≥-5.941, *q*_O_<-0.371, *d*_2_≥2.037, *r*_+1_^min^≤2.093 |
|  | | |
| *q*(CO_2_) minimization with Sabatier principle constraint | | |
| -0.1 | 82 | *IP*_max_≥-5.941, *VBM*≤-5.0995, Δφ≥0.7326, *r*_-1_^min^≤1.652, *α*_O_≤3.11045 |
| -0.2 | 39 | *EA*_max_≤0.005, *EA*_max_≥-0.4945, Δφ≥0.7326, *r*_-1_^min^≤1.666, *E*_form_≤0.085 |
| -0.3 | 15 | *C*_6_^min^>485.0, *c*_min_≤-9.58, *kurt*≥3.1545, *E*_form_<0.062 |

**
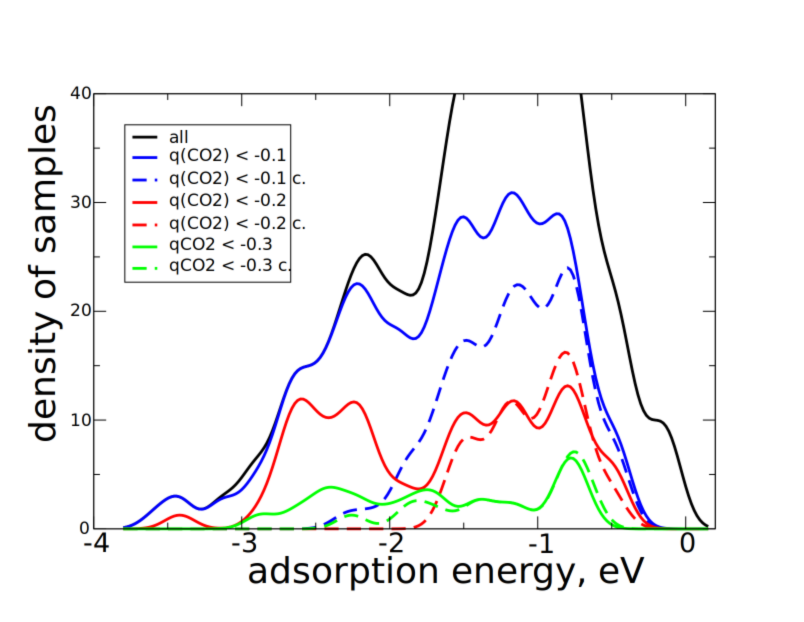
**

**Supplementary Figure 7**. The distribution of adsorption energies in the subgroups of samples with larger absolute values of Hirshfeld charge of an adsorbed CO_2_.

**3. Difference in Hirshfeld charges of C and O atoms in an adsorbed CO_2_.** This property indicates the ionicity of a C-O bond. Larger ionicity is expected to correlate with the reactivity in reactions with electrophilic or nucleophilic agents. The calculated CO_2_ gas-phase value of the charge difference is 0.44 *e*. It lies within the range of the data for adsorbed CO_2_, namely, 0.38-0.52 *e* (Supplementary Figure 8, left). We have done the SGD search of subgroups with positive shift with three cutoffs: 0.45, 0.47, and 0.48 *e*. Obtained subgroups are shown in Supplementary Table 7.

In the case when no Sabatier principle constraint was accounted for, all subgroups contain the samples for which strong carbonation is observed (Supplementary Figure 8). There is a certain overlap with reduced OCO subgroups. For example, the subgroup *q*(C)-*q*(O) > 0.45*e* contains 18 common samples (49%) with OCO < 130° subgroups. The subgroups obtained with Sabatier principle constraint also partially overlap with constrained OCO subgroups – 10 common samples for *q*(C)-*q*(O) > 0.45*e* with OCO > 128° and 16 with OCO > 130° subgroups. Even larger relative overlap is obtained with *l*(C-O) > 1.30 Å subgroup – 16 samples (67%). Smaller size constrained subgroups with 0.47 and 0.48*e* cutoffs have also about 60% common samples with *l*(C-O) > 1.30 Å subgroup.

**Supplementary Table 7.** The subgroups obtained for SGD maximization of the difference in Hirshfeld charges of C and O atoms.

| threshold | size | subgroup |
| --- | --- | --- |
| *q*(CO_2_) minimization without Sabatier principle constraint | | |
| 0.45 | 37 | *W<*5.5255, *PC*>-7.53, *C*_6_<=11.1305, skew>=-2.1615 |
| 0.47 | 9 | *L*_max_>-1.2615, φ_1.4_>=1.2116, *α*_O_<-0.1558 |
| 0.48 | 8 | Δφ>=0.996, *M*>-5.1865, *q*_max_>=0.5483 |
|  | | |
| *q*(CO_2_) minimization with Sabatier principle constraint | | |
| 0.45 | 24 | *EN*_min_<=3.633, *EN*_max_>-3.039, *PC*>=-8.895, *r*_HOMO_^min^<=1.337 |
| 0.47 | 7 | *C*_6_^max^>1440.5, *C*_6_^min^<=830.5, *Q*_5_<=0.7952, *d*_2_>2.217, *r*_HOMO_^max^>=1.344 |
| 0.48 | 7 | *C*_6_^max^>1440.5, *C*_6_^min^<=830.5, *Q*_5_<=0.7952, *d*_2_>2.217, *r*_HOMO_^max^>=1.344 |


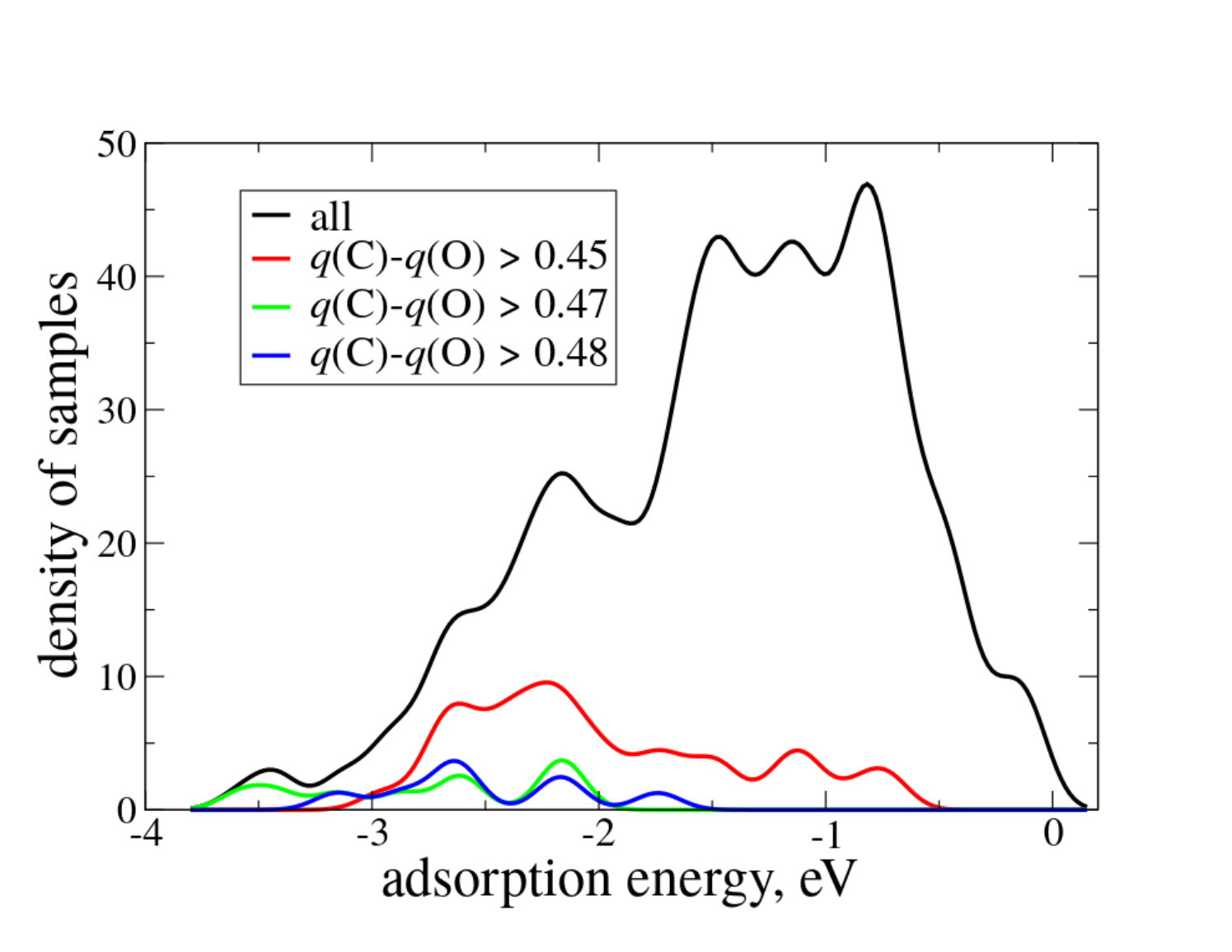


**Supplementary Figure 8**. The distribution of adsorption energies in the subgroups with increased *q*(C)-*q*(O) without Sabatier principle constraint.

**4. Difference of Hirshfeld charges on O-atoms of an adsorbed CO_2_.** As we show, the elongated C-O bonds are observed when the CO_2_ molecule is adsorbed in an asymmetric position, so that one oxygen atom is bonded with a surface cation and the other one is protruding. In these cases, the two O-atoms have nonequivalent chemical surroundings. Correspondingly, the difference of Hirshfeld charges on CO_2_ oxygens, Δ*q*(O), is expected to indicate this asymmetry. The SGD with the absolute Δ*q*(O) as target property was performed with maximizing this difference with the quality function (1) in the main text. The next thresholds have been considered: 0.01, 0.02 and 0.03 *e*. Obtained subgroups are summarized in Supplementary Supplementary Table 8.

The analysis of their populations shows significant overlap with the subgroups for elongated C-O bond distances. For instance, there are 21 common samples in *l*(C-O) > 1.3 Å subgroup and the subgroup with Δ*q*(O) > 0.01 *e*, 81% and 78% of respective populations. The overlap for constrained and unconstrained with Sabatier principle subgroups is 100%. The samples in Δ*q*(O) subgroups with larger thresholds are mostly the same as ones in Δ*q*(O) > 0.01 *e* subgroup. So, we conclude that the difference of Hirshfeld charges on CO_2_ oxygen atoms basically reproduces the C-O bond length indicator.

**Supplementary Table 8.** The subgroups obtained for SGD maximization of Hirshfeld charges difference on O-atoms in an adsorbed CO_2_.

| threshold | size | subgroup |
| --- | --- | --- |
| maximization of Δ*q*(O) without Sabatier principle constraint | | |
| 0.01 | 25 | Δφ>=0.596, *PC*<=-7.207, *d*_2_>2.217, *r*_-1_^min^<=1.1235 |
|  | | |
| maximization of Δ*q*(O) with Sabatier principle constraint | | |
| 0.01 | 26 | *EA*_max_<=0.005, *c*_min_<=-5.849*, d*_2_*>*2.217, *q*_min_*<=*0.51 |
| 0.02 | 19 | *C*_6_^max^>=580.5, *EA*_max_<=0.0375, φ_1.4_>=0.66415, *IP*_min_<=6.4695, *α*_max_>90.75, *C*_6_>=9.025 |
| 0.03 | 11 | *IP*_max_>-5.5225, *α*_min_<=60.55, *d*_1_<=1.9585, *M*>=-7.555, *α*_O_>=0.2268 |

**Supplementary References**

1. Blum, V. et al. Ab initio molecular simulations with numeric atom-centered orbitals. *Comput*. *Phys. Comm.* **180**, 2175-2196 (2009).
2. Perdew, J. P., Burke, K. & Ernzerhof, M. Generalized gradient approximation made simple. *Phys. Rev. Lett.* **77**, 3865–3868 (1997).
3. Perdew, J. P. et al. Restoring the density-gradient expansion for exchange in solids and surfaces. *Phys. Rev. Lett.* **100**, 136406 (2008).
4. Tkatchenko, A. & Scheffler, M. Accurate molecular van der Waals interactions from ground-state electron density and free-atom reference data. *Phys. Rev. Lett.* **102**, 073005 (2009).
5. Ceperley, D. M. & Alder, B. J. Ground state of the electron gas by a stochastic method. *Phys. Rev. Lett*. **45**, 566–569 (1980).
6. Hammer, B., Hansen, L. B. & Nørskov, J. K. Improved adsorption energetics within density-functional theory using revised Perdew-Burke-Ernzerhof functionals. *Phys. Rev. B* **59**, 7413-7421 (1999).
7. Blaylock, D. W., Ogura, T., Green, W. H. & Beran, G. J. O. Computational investigation of thermochemistry and kinetics of steam methane reforming on Ni(111) under realistic conditions. *J. Phys. Chem. C* **113**, 4898-4908 (2009).
8. Peterson, A. A., Abild-Pedersen, F., Studt, F., Rossmeisl & J., Nørskov, J. K. How copper catalyzes the electroreduction of carbon dioxide into hydrocarbon fuels. *Energy Environ. Sci.* **3**, 1311-1315 (2010).
9. Neugebauer, J. & Scheffler, M. Adsorbate-substrate and adsorbate-adsorbate interactions of Na and K adlayers on Al(111). *Phys. Rev. B.* **46**, 16067-16080 (1992).
10. Solis, B. H. et al. Initial stages of CO_2_ adsorption on CaO: a combined experimental and computational study. *Phys. Chem. Chem. Phys.* **19**, 4231-4242 (2017).
11. Wang, Y. et al. CO_2_ activation by ZnO through the formation of an unusual tridentate surface carbonate. *Angew. Chem. Int. Ed.* **46**, 5624–5627 (2007).
12. Xia, X., Strunk, J., Busser, W., Naumann d’Alnoncourt, R. & Muhler, M. Probing the surface heterogeneity of polycrystalline zinc oxide by static adsorption microcalorimetry. 1. The influence of the thermal pretreatment on the adsorption of carbon dioxide. *J. Phys. Chem. C* **112**, 10938–10942 (2008).
13. Bučko, T., Lebegue, S., Angyan, J. G. & Hafner, J. Extending the applicability of the Tkatchenko-Scheffler dispersion correction via iterative Hirshfeld partitioning. *J. Chem. Phys.* **141**, 034114 (2014).
14. Meixner, D., Arthur, D. & George, S. Kinetics of desorption, adsorption, and surface diffusion of CO_2_ on MgO(100). *Surf. Sci.* **261**, 141–154 (1992).
15. Mazheika, A. & Levchenko, S. V. Ni Substitutional Defects in bulk and at the (001) surface of MgO from first-principles calculations. *J. Phys. Chem. C* **120**, 26934–26944 (2016).
16. <http://nomad-repository.eu/>
17. Hinuma, Y., Hayashi, H., Kumagai, Y., Tanaka, I. & Oba, F. *Phys. Rev. B* **96**, 094102 (2017).
18. Hirshfeld, F. L. Bonded-atom fragments for describing molecular charge densities. *Theor. Chim. Acta*, **44**, 129–138 (1977).
